# Anti‐PD‐1/Her2 Bispecific Antibody IBI315 Enhances the Treatment Effect of Her2‐Positive Gastric Cancer through Gasdermin B‐Cleavage Induced Pyroptosis

**DOI:** 10.1002/advs.202303908

**Published:** 2023-08-16

**Authors:** Wu Lin, Yingzi Zhang, Yan Yang, Ben Lin, Mengjia Zhu, Jinling Xu, YiRan Chen, Weiwei Wu, Bingliang Chen, Xiangliu Chen, Jin Liu, Haohao Wang, Fei Teng, Xiongfei Yu, Haiyong Wang, Jun Lu, Quan Zhou, Lisong Teng

**Affiliations:** ^1^ Department of Surgical Oncology The First Affiliated Hospital Zhejiang University School of Medicine Hangzhou Zhejiang 310003 China; ^2^ Department of Colorectal Surgery and Oncology (Key Laboratory of Cancer Prevention and Intervention, China National Ministry of Education, Key Laboratory of Molecular Biology in Medical Sciences, Zhejiang Province, China) The Second Affiliated Hospital, Zhejiang University School of Medicine Hangzhou Zhejiang 310009 China; ^3^ Zhejiang Provincial Clinical Research Center for CANCER Hangzhou Zhejiang 310009 China; ^4^ Cancer Center of Zhejiang University Hangzhou Zhejiang 310009 China; ^5^ College of Medicine Jiaxing University Jiaxing Zhejiang 314001 China; ^6^ Department of Drug Discovery Innovent Biologics (Suzhou) Co. Suzhou Jiangsu 215000 China; ^7^ Institute of Immunology, Department of Surgical Oncology of The First Affiliated Hospital Zhejiang University School of Medicine Hangzhou Zhejiang 310058 China

**Keywords:** bispecific antibodies, GSDMB, Her2, immunotherapy, pyroptosis

## Abstract

The majority of patients with human epidermal growth factor receptor 2 (Her2)‐positive gastric cancer develop refractory to Her2‐targeted therapy, where upregulation of immune checkpoints plays an essential role. Herein, a recombinant fully human IgG1 bispecific antibody IBI315 targeting both PD‐1 and Her2 is developed  and its antitumor efficacy as well as the underlying mechanism is investigated. IBI315 crosslinks the physical interaction between Her2‐positive tumor cells and PD‐1‐positive T cells, resulting in significantly enhanced antitumor effects compared to each parent antibody or their combination, both in vitro and in vivo mouse tumor models reconstituted with human immune cells using patient‐derived xenografts and organoids. Moreover, IBI315 treatment also induces the recruitment and activation of immune cells in tumors. Mechanistically, IBI315 triggers gasdermin B (GSDMB)‐mediated pyroptosis in tumor cells, leading to the activation and recruiments of T cells. The activated T cells secret IFNγ, enhancing GSDMB expression and establishing a positive feedback loop of T cell activation and tumor cell killing. Notably, GSDMB is found to be elevated in Her2‐positive gastric cancer cells, providing a rationale for IBI315's efficacy. IBI315 is supported here as a promising bispecific antibody‐based immunotherapy approach for Her2‐positive gastric cancer in preclinical studies, broadening the therapeutic landscape of this patient population.

## Graphical abstract

IBI315, a bispecific antibody targeting PD‐1 and Her2, enhances antitumor effects through tumor‐T cell interaction. It outperforms parent antibodies in vitro and in vivo. Mechanistically, IBI315 triggers gasdermin B (GSDMB)‐mediated pyroptosis, activating T cells and establishing a positive feedback loop. Elevated GSDMB in Her2‐positive gastric cancer cells supports IBI315's efficacy, highlighting its potential for refractory Her2‐positive gastric cancer immunotherapy.

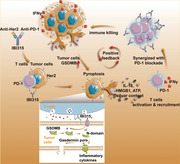

## 1 Introduction

Therapy with trastuzumab represents the standard of care therapy for patients with human epidermal growth factor receptor 2 (Her2)‐positive gastric cancers.^[^ ^1^ ^]^ However, ≈70% of patients with Her2‐positive gastric cancer eventually develop resistance to trastuzumab and most patients who initially respond to trastuzumab often develop resistance or relapse within one year.^[^ ^2^, ^3^ ^]^ In addition to the downregulation of cell surface Her2 or mutations in the Her2‐mediated PI3‐kinase/Protein kinase B(AKT） signaling pathways,^[^ ^4^ ^]^ upregulation of immunosuppressive molecules such as programmed cell death protein ligand‐ 1 (PD‐L1) and CD73 are associated with trastuzumab resistance.^[^ ^5^, ^6^ ^]^ Therefore, therapies that combine the targeting of Her2 and these immunosuppressive molecules are of significant clinical interest. Consistently, the KEYNOTE‐811 study confirmed that adding pembrolizumab to trastuzumab and chemotherapy achieved an objective response rate (ORR) of 74.4% and markedly reduced tumor size in patients with Her2‐positive gastric cancer/gastroesophageal junction adenocarcinoma.^[^ ^7^ ^]^ Therefore, the targeting of Her2 and PD‐1 has huge prospects for clinical application to overcome the resistance of Her2‐positive gastric cancer to trastuzumab.

Bispecific antibodies (BsAbs) comprise two distinct antigen‐targeting domains and can be used in place of a combination of two monoclonal antibodies (mAbs).^[^ ^8^, ^9^ ^]^ IBI315 is a recombinant fully human IgG1 PD‐1/Her2 BsAbs jointly developed by Innovent Biopharmaceutical and Hanmi Pharmaceutical. The parental antibodies of IBI315 are trastuzumab and sintilimab (an anti‐PD1 antibody), Fc mutation was introduced to form heterodimers (Patent No.: US20190367633). The preliminary results of the Phase Ia clinical study of IBI315 were reported at the 2021 Annual Meeting of the Chinese Society of Clinical Oncology. As of May 25, 2021, the study enrolled 27 patients with Her2‐expressing advanced solid tumors who failed in standard therapy and received 7 dose levels of 0.03 mg kg^−1^ QW (every week) to 15 mg kg^−1^ Q3W (every 3 weeks), respectively. Dose‐limiting toxicity and the maximum tolerated dose were not reached. A total of 15 patients who received the predicted effective dose (≥1 mg kg^−1^) underwent at least one tumor assessment yielding an ORR of 20%. In the meantime, biomarker analysis yielded consistent results to clinical efficacy; peripheral immune cell proliferation and activation were higher in responders to treatment. Previous studies have substantiated that PD‐1/Her2 BsAbs have dual blocking activities of Her2 and PD‐1 through in vivo/in vitro experiments and yield a killing effect on Her2‐positive cancers through antibody‐dependent cytotoxicity.^[^ ^10^ ^]^ In addition, PD‐1/Her2 bispecific antibodies can crosslink Her2‐positive tumor cells and PD‐1‐positive T cells to immune synapses, thereby directing tumor cell killing without antigen recognition, providing a new approach with potential therapeutic benefits that mAbs cannot provide.^[^ ^11^, ^12^, ^13^, ^14^, ^15^ ^]^

Here, we investigated the function and mechanisms of IBI315 in the treatment of Her2‐positive gastric cancers in patient‐derived xenografts (PDXs) and patient‐derived organoids (PDOs). We found that the potent antitumor effect of IBI315 was dependent on the pyroptosis of tumor cells mediated by activation of gasdermin B (GSDMB). Interestingly, the pyroptosis of tumor cells induced by IBI315 leads to recruitment and activation of immune cells in tumors. Notably, the activated T cells secrete Interferon‐gamma (IFNγ), which in turn enhances GSDMB expression, thus establishing a positive feedback loop that augments T cell activation and facilitates efficient tumor cell elimination. Remarkably, an upregulation of GSDMB expression was observed in Her2‐positive gastric cancer cells, thereby substantiating the basis for the effectiveness of IBI315. This investigation provides support for IBI315 as a promising bispecific antibody‐based immunotherapeutic strategy in preclinical evaluations, thereby broadening the therapeutic landscape for Her2‐positive gastric cancer patients.

## 2 Results

### 2.1 IBI315 Induces the Association of PD‐1 and Her2 and Mediates T Cell‐Mediated Cytotoxicity against Her2‐Positive Gastric Cancer Cells

**Figure 1:**
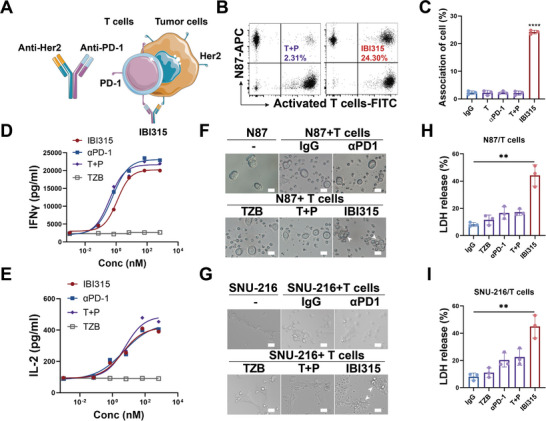
IBI315 induces the association of PD‐1 and Her2 and mediates T cell‐mediated cytotoxicity against Her2‐positive gastric cancer cells. A–C) N87 cells were labeled with cell tracker Deep Red and activated T cells were labeled with Carboxyfluorescein succinimidyl ester (CFSE). They were cocultured at a 1:1 ratio and treated with IBI315, its parent antibody, or their combination for 30 min. The association rate between N87 cells and activated T cells was analyzed using flow cytometry. A) A schematic model depicts the association of IBI315 with PD‐1 on T cells and Her2 on tumor cells. B) Representative flow cytometry images show the association of N87 cells (stained with cell tracker Deep Red) and activated T cells (stained with CFSE) treated with T+P or with IBI315. C) Quantification of associated cells (cells in the upper right quadrant of the scatter plot) with the indicated treatments is presented based on flow cytometry analysis. D,E) The impact of IBI315, parental PD‐1 antibody, parental trastuzumab, and the combination of parental drugs on the secretion of D) IFNγ and E) IL‐2 by human T cells was investigated using a mixed lymphocyte reaction (MLR). F–I) Her2‐positive gastric cancer cells (N87 and SNU‐216) were treated with IBI315, its parent antibody, or their combination for 24 h in the presence of T cells. Representative images of F) the N87/T cell and G) SNU‐216/T cell coculture systems are shown, with the white arrow indicating T cells associated with tumor cells. Quantification of tumor cell death, measured by LDH release in H) the N87/T cell and I) SNU‐216/T cell coculture systems, is presented for the indicated treatments. Scale bar 50 µm. All statistical results were obtained from three independent experiments and expressed as means ± SEM. Abbreviations: TZB: trastuzumab, αPD1: anti‐PD1 antibody (sintilimab), T+P: trastuzumab+sintilimab. Statistical significance is indicated as **P < 0.01 and ***P < 0.001.

We tested the binding affinity of IBI315 to Her2 and PD‐1 by the surface plasmon resonance (SPR) experiments. The SPR data showed that IBI315 had high binding affinity for both PD‐1 and Her2, with an equilibrium dissociation constant (*K*_D_) value of 1.17 × 10^−9^ and 3.55 × 10^−10^ m, respectively. Compared to its parental antibodies, IBI315 exhibited a higher binding affinity for Her2 than Trastuzumab, with a 1.2‐fold higher association rate constant (*k*_on_) and 1.8‐fold lower dissociation rate constant (*k*_off_). IBI315 also showed a 1.5‐fold higher association rate constant and 1.7‐fold lower dissociation rate constant for PD‐1 than Sintilimab, resulting in a 1.1‐fold lower *K*_D_ value (**Table** **1**). These findings suggested that IBI315 is a potent bispecific antibody with higher binding affinity for both PD‐1 and Her2 compared to its parental antibodies. To evaluate whether IBI315 facilitates cell bridging by binding to each receptor, we fluorescently labeled activated T cells (green) and N87 (red) cells with different dyes and treated them with the indicated antibodies, followed by flow cytometry analysis. IBI315 addition induced association of activated T cells and N87 cells, yielding a doublet rate of 24.10% ± 0.55%, whereas no association was observed with either its parental mAbs or the parental two‐drug combination (**Figure** **1**B,C). To further investigate the effect of IBI315 on T cell activation mediated by PD‐1 blockade, we conducted a mixed lymphocyte reaction (MLR) experiment, and the results indicated that IBI315 exhibits a similar potent activating effect on human T cells as its parental PD‐1 antibody (Figure 1D,E). In addition, we observed elevated PD‐1 expression on T cells following coculture with gastric cancer cells (Figure S1, Supporting Information). Given that IBI315 could crosslink PD‐1 on T cells and Her2 on tumor cells, we hypothesized that IBI315 might enhance tumor cell killing by bringing T cells into the vicinity of tumor cells and possibly inducing immune synapse formation.^[^ ^10^ ^]^ We established a coculture system of tumor cells and activated T cells as previously described (Figure S2, Supporting Information).^[^ ^16^ ^]^ In the coculture system, IBI315 demonstrated significantly more potent cytotoxicity (*p* < 0.01, Figure 1H,I and Figure S3, Supporting Information) against Her2‐positive gastric cancer cells compared with the parental mAbs, trastuzumab, sintilimab or the parental two‐drug combination. We also observed an association between T cells and tumor cells in the coculture system with the addition of IBI315 (Figure 1F,G). In addition, IBI315 retains the antibody‐dependent cellular cytotoxicity (ADCC) activity of its parental trastuzumab. When peripheral blood mononuclear cells (PBMCs) was used as effector cells, IBI315 still demonstrated the strongest tumor‐killing activity compared to its parental antibody (Figure S4, Supporting Information). These results demonstrated that IBI315 could induce association of PD‐1 and Her2 and mediated T cell‐mediated cytotoxicity against Her2‐positive gastric cancer cells. We also indicated that IBI315 exhibits potent killing activity against Her2‐positive breast cancer cells (Figure S5, Supporting Information).

**Table 1:** Binding kinetics to human PD‐1 and Her2 by Biacore

Antibody	Analyte	*K*_on_ [1 Ms^−1^]	*K*_off_ [1 s^−1^]	*K*_D_ [m]
IBI315 (anti‐hPD‐1/Her2)	PD‐1 human	2.04 × 10^5^	2.38 × 10^−4^	1.17 × 10^−9^
	Her2 human	3.78 × 10^5^	1.34 × 10^−4^	3.55 × 10^−10^
Trastuzumab (anti‐hHer2)	Her2 human	3.81 × 10^5^	2.22 × 10^−4^	5.83 × 10^−10^
Sintilimab (anti‐hPD‐1)	PD‐1 human	2.03 × 10^5^	2.80 × 10^−4^	1.38 × 10^−9^

In order to evaluate the safety of IBI315, we conducted an analysis to assess its potential to induce cytokine release syndrome. Our investigation revealed that IBI315 exhibited negligible induction of immune cell secretion of Interleukin‐2 (IL‐2), IL‐4, IL‐6, IL‐10, TNFα, and IL‐17A (Figure S6, Supporting Information). These results suggested that IBI315 elicits a minimal cytokine release syndrome, highlighting its favorable safety profile.

### 2.2 IBI315 Exhibits Potent Antitumor Efficacy in Her2‐Positive Gastric Cancers and Organoids

**Figure 2:**
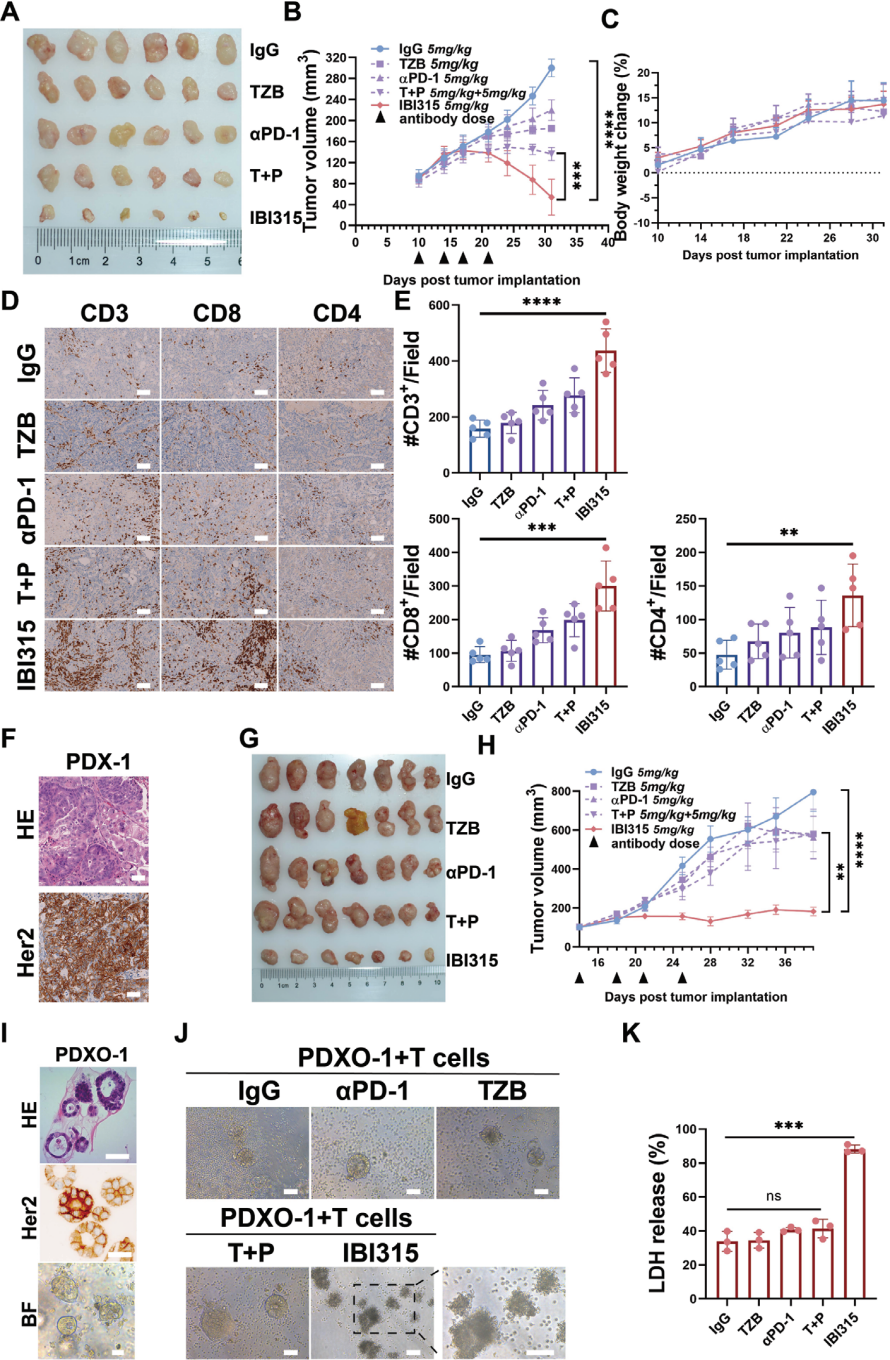
IBI315 exhibits potent antitumor efficacy in Her2‐positive gastric cancers and organoids. A–H) Human immune cell‐reconstituted NOG mice bearing N87 tumors or patient‐derived xenografts (PDX‐1) were subjected to treatment with IBI315, its parent antibody, or their combination. A) Photographic documentation of N87 tumors on day 31 and B) the corresponding average tumor volumes were obtained. C) Additionally, the change in body weight for each treatment group was monitored. D) Immunohistochemical analysis revealed representative images of CD3^+^, CD8^+^, and CD4^+^ T cell infiltration within N87 tumors for each treatment group, E) which were further quantified. F) Histopathological examination of PDX‐1 included H&E and Her2 staining, along with G) photographic documentation on day 39 and H) assessment of average tumor volumes. I–K) A coculture system of organoid PDXO‐1 and T cells was established to evaluate tumor cell death induced by IBI315, its parent antibody, or their combination, as determined by measuring LDH release. I) Histological analysis of organoid PDXO‐1 included H&E staining, Her2 staining, and bright field (BF) imaging. J) Representative images of the PDXO‐1/T cells coculture system under indicated treatments were captured, with a specific focus on IBI315‐treated PDXO‐1, showcasing the association between T cells and PDXO‐1. K) The extent of PDXO‐1 cell death was quantified by measuring LDH release after a 24‐hour incubation with T cells under indicated treatments. Scale bar 50 µm. All statistical results were obtained from three independent experiments and expressed as means ± SEM. Abbreviations: TZB: trastuzumab, αPD1: anti‐PD1 antibody(sintilimab), T+P: trastuzumab+sintilimab. Statistical significance is indicated as **P < 0.01, ***P < 0.001, ****P < 0.0001.

Next, we evaluated the therapeutic efficacy of IBI315 in vivo using Her2‐positive humanized tumor xenograft mouse models established as reported previously.^[^ ^16^ ^]^ Humanized mice bearing N87 tumors (*n* = 30) or Her2‐positive PDX (**Figure** **2**F, PDX‐1; *n* = 35) were randomly divided into five treatment groups, and IBI315, IgG (control), trastuzumab, sintilimab or combined trastuzumab and sintilimab were injected intraperitoneally. Analysis of tumor volume showed that IBI315 exhibited more potent antitumor efficacy compared to the parental sintilimab and trastuzumab combination or respective monotherapies in the N87 tumor model and PDX‐1 model (Figure 2A,B,G,H). Notably, no significant effect on the body weight decrease of mice was observed (Figure 2C). In the N87 tumor model, the reduction rate reached 117% compared to the control (54.1 ± 34 vs 300.1 ± 17 mm^3^) in the IBI315 treatment group. In the PDX‐1 model, no significant tumor growth was observed with a reduction of 77% (182.2 ± 14 vs 795.4 ± 235 mm^3^) in the IBI315 treatment group, whereas the parental mAbs or the parental two‐drug combination treatment groups exhibited no significant difference in tumor growth rate compared to the control group (Figure 2G,H). We next assessed whether the potent antitumor effect of IBI315 is due to higher immune cell infiltration and quantified the CD3^+^, CD8^+^, and CD4^+^ tumor‐infiltrating lymphocytes in the xenografts. Indeed, the IBI315‐treated tumor exhibited the highest infiltration of CD3^+^, CD8^+^, and CD4^+^ lymphocytes compared to the control group, parental mAbs groups, or the parental two‐drug combination group in both N87 tumor model and PDX‐1 model (Figure 2D,E and Figure S7, Supporting Information).

To assess whether IBI315 could enhance the cytotoxic effect of T cells against tumor cells, we used a PDO and T cells coculture system. Given that PDO was derived from PDX‐1, it was referred to as organoid from patient‐derived tumor xenograft‐1 (PDXO‐1) in the text below (Figure 2I). The effect of each treatment on PDXO‐1 was consistent with the effect of PDX‐1 in humanized mice (IBI315 exhibited two to threefold effectiveness than control, while parental mAbs or the parental two‐drug combination group showed no significant difference with control; Figure 2K). Importantly, the addition of IBI315 induced the association of T cells and PDXO‐1 (Figure 2J). These results showed that IBI315 yielded potent antitumor efficacy in Her2‐positive gastric cancers and organoids.

### 2.3 IBI315 Induces Pyroptosis in Her2‐Positive Gastric Cancer Cells in the Presence of T Cells

**Figure 3:**
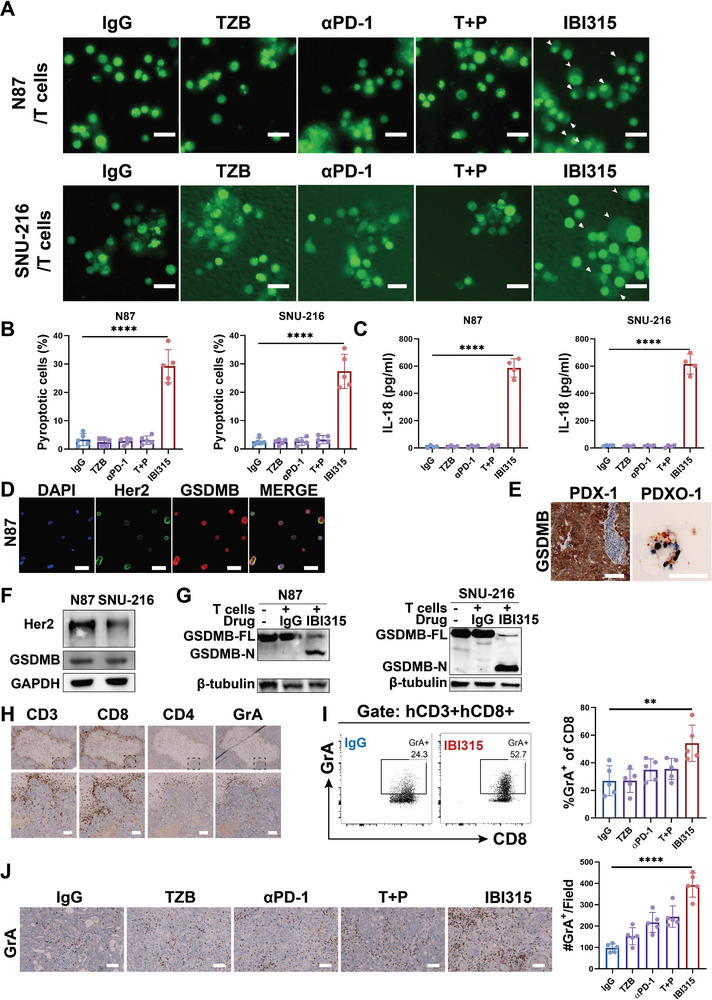
IBI315 induces pyroptosis in Her2‐positive gastric cancer cells in the presence of T cells. A–C) N87 or SNU‐216 cells were prelabeled with CFSE and cocultured with T cells in the presence of IBI315, its parent antibody, or their combination for 6 h. Fluorescent images were captured to illustrate the morphological changes of tumor cells, and the levels of IL‐18 in the supernatant of the tumor cell/T cell coculture system were measured. A) Representative images of N87 or SNU‐216 cells with arrowheads indicating pyroptotic cells. B) The quantification of pyroptotic cells following treatment with the indicated antibodies is presented in a column chart. C) The levels of IL‐18 in the supernatant of the tumor cell/T cell coculture system under the indicated treatments are shown. D–G) The expression of GSDMB in N87, SNU‐216 cells, and PDXO‐1 was examined and its cleavage was induced by IBI315 in the presence of T cells for 6 h. D) Coexpression of Her2 (green) and GSDMB (red) in N87 cells was visualized by fluorescent staining. E) GSDMB expression in PDX‐1 and PDXO‐1 was assessed by immunohistochemical staining. F) Immunoblotting was performed to evaluate the expression of Her2 and GSDMB in N87 and SNU‐216 cells. G) Immunoblotting was also conducted to assess GSDMB cleavage in the tumor cell/T cell coculture system treated with IgG or IBI315 for 6 h. H–J) The expression pattern of granzyme A, an enzyme involved in GSDMB cleavage‐induced pyroptosis, was evaluated in tumor‐infiltrating lymphocytes (TILs) from tumors treated with IBI315, its parent antibody, or their combination (following the same administration protocol as in Figure 2). H) Immunohistochemical staining revealed the expressions of CD3, CD8, CD4, and granzyme A at the same site in TILs of N87 tumors treated with IBI315. I) The PDX‐1 tumors were digested into single cells and the TILs were analyzed. The proportion of granzyme A^+^ cells in CD8^+^ TILs was determined by flow cytometry, with representative graphs depicting IgG or IBI315 treatment in PDX‐1, and their quantification in each group. J) Immunohistochemical staining images and quantification of granzyme A^+^ TILs in N87 tumors for each treatment group. Scale bar 100 µm. All statistical results represent the means ± SEM of three independent experiments. Abbreviations: TZB: trastuzumab, αPD1: anti‐PD1 antibody (sintilimab), T+P: trastuzumab+sintilimab. Statistical significance is indicated as **P < 0.01, ****P < 0.0001.

As the potent antitumor efficacy to Her2‐positive gastric cancer we have seen, we want to further investigate the mechanism. In the N87 cells/T cells coculture system, N87 cells treated with IBI315 presented cell swelling‐like morphological changes similar to cell pyroptosis (Figure S8, Supporting Information). To investigate whether this phenomenon is unique to the IBI315‐treated group, morphological changes in N87 and Seoul National University‐216 (SNU‐216) cells after treatment with IBI315, parental mAbs, parental two‐drug combination and blank IgG were compared in the presence of T cells. Only the IBI315‐treated cells showed significant swelling, representing the characteristic cell morphological changes in cell pyroptosis (**Figure** **3**A,B). Importantly, we observed a large amount of IL‐18, the proinflammatory cytokines released from pyroptotic cells, in the supernatant of the IBI315 treatment group (Figure 3C). This result indicated that IBI315 could mediate tumor cell pyroptosis in the presence of T cells.

The occurrence of pyroptosis depends on the activation and cleavage of Gasdermin, which has six members. The Cancer Genome Atlas (TCGA) database screenings showed that GSDMB, ‐D, and ‐E were expressed in gastric cancers (Figure S9A, Supporting Information). We found that N87 cells only expressed GSDMB after screening these three GSDM proteins (Figure S9B, Supporting Information). Next, we verified that GSDMB was expressed in the abovementioned cells and models, namely SNU‐216 cells, Her2‐positive gastric cancer PDX‐1 and PDXO‐1(Figure 3D–F). Most importantly, when N87 and SNU‐216 cells were treated with IBI315 in the presence of T cells, GSDMB cleavage was found, and the N‐terminal fragment (which is the executor of cell pyroptosis) in both cell lines was increased compared with the IgG‐treated group (Figure 3G). Subsequently, we sought to investigate what cleaves GSDMB in tumor cells in the presence of T cells. Shao and co‐workers previously demonstrated that the induction of pyroptosis in target cells is mediated by granzyme A (GZMA), which is secreted by cytotoxic lymphocytes and cleaves GSDMB.^[^ ^17^ ^]^ This finding provided valuable insights for our study. Subsequent investigations have indicated that the N‐terminal fragment of GSDMB generated by cleavage from Neutrophil Elastase or caspases is incapable of inducing pyroptosis, while cleavage by granzyme A specifically generates an N‐terminal fragment that possesses the ability to induce pyroptosis.^[^ ^18^ ^]^ To further investigate the role of granzyme A, we performed immunostaining on the N87 tumor tissue (following the same administration protocol as in Figure 2A,B) and observed alterations in the expression of infiltrating immune cell‐derived granzyme A that were consistent with treatment response (Figure 3H,J). Furthermore, we found that the IBI315‐treated group exhibited higher expression of granzyme A in CD8‐positive T lymphocytes infiltrating the PDX‐1 tumor (Figure 3I). These findings suggest that granzyme A may also contribute to the cleavage of GSDMB in our experimental system.

### 2.4 The Tumoricidal Effect of IBI315 Depends on GSDMB‐Cleavage‐Induced Pyroptosis

**Figure 4:**
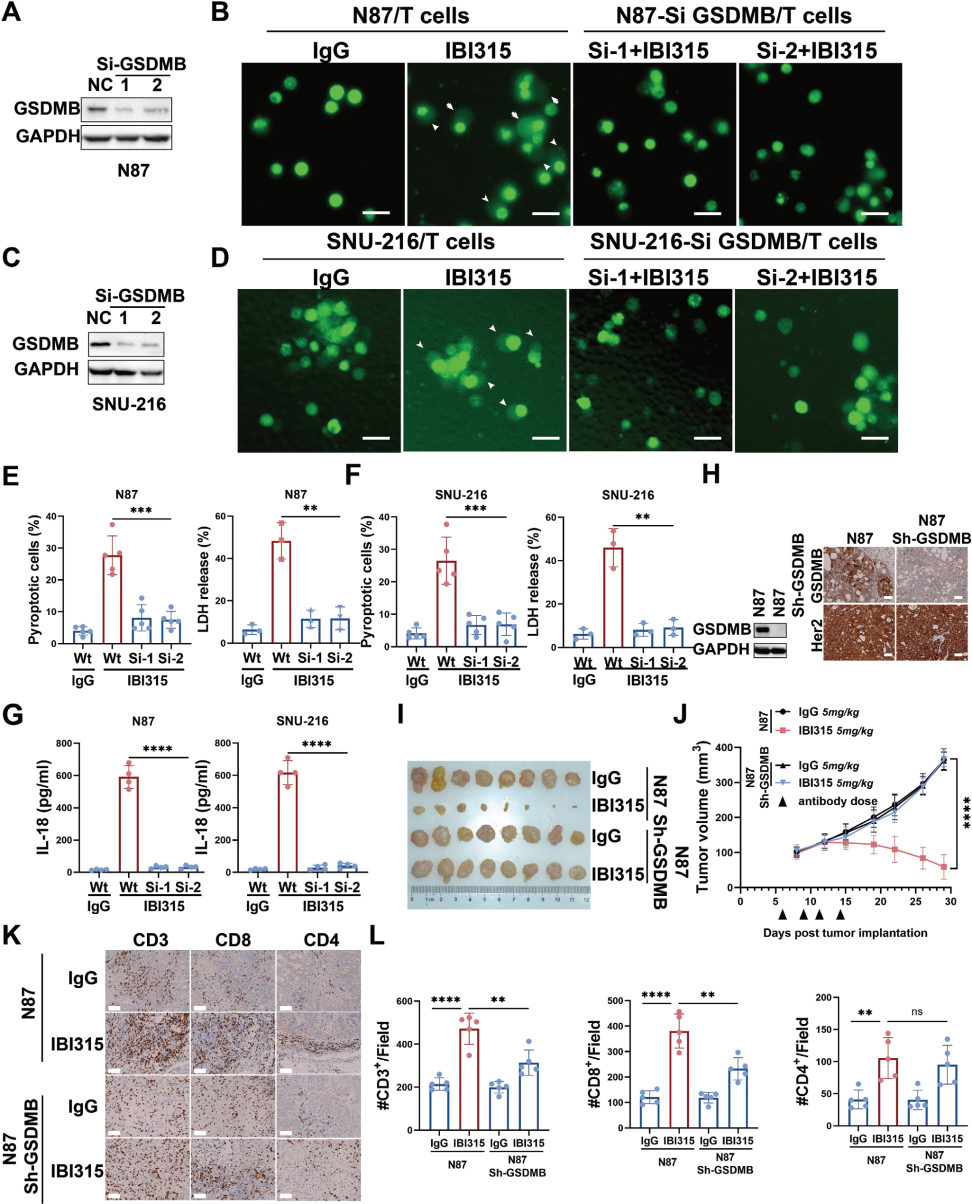
The tumoricidal effect of IBI315 depends on GSDMB‐cleavage‐induced pyroptosis. GSDMB expression in N87 and SNU‐216 cells was downregulated using siRNA (siGSDMB‐1 and siGSDMB‐2). The cells were then labeled with CFSE and cocultured with T cells in the presence of either IBI315 or IgG. Fluorescent imaging was performed to visualize the morphological changes associated with pyroptosis in tumor cells with or without GSDMB knockdown (±GSDMB) and the levels of IL‐18 in the supernatant of the tumor cell (±GSDMB)/T cell coculture system were measured. Anti‐GSDMB immunoblotting was conducted in A) N87 and C) SNU‐216 cells before and after treatment with siGSDMB‐1 and siGSDMB‐2 to evaluate the efficacy of siRNA. Representative images of B) prelabeled N87 or D) SNU‐216 cells in the tumor cell (±GSDMB)/T cell coculture system were captured, with arrowheads indicating pyroptotic cells. E,F) Quantification of pyroptotic cells and LDH release was performed. G) The levels of IL‐18 in the supernatant of the tumor cell (±GSDMB)/T cell coculture system under the indicated treatments were measured. H) GSDMB knockdown in N87 cells was achieved using shRNA (N87 Sh‐GSDMB). NOG mice reconstituted with human immune cells and bearing N87 or N87 Sh‐GSDMB tumors were treated with IBI315 or IgG. Anti‐GSDMB immunoblotting was conducted in N87 cells, and GSDMB and Her2 staining were performed in N87 tumors to confirm the efficacy of shGSDMB. I) Photographic documentation of N87 tumors on day 29 and J) the corresponding average tumor volumes were obtained. K) Immunohistochemical analysis revealed representative images of CD3^+^, CD8^+^, and CD4^+^ T cell infiltration within N87 tumors for each treatment group, L) which were further quantified. All statistical results were obtained from three independent experiments and expressed as means ± SEM. Scale bar 100 µm. Statistical significance is indicated as ns *P* > 0.05, **P < 0.01, ***P < 0.001, ****P < 0.0001.

To explore whether pyroptosis induced by GSDMB cleavage is the main mechanism underlying the antitumor effect of IBI315, we knocked down the expression of GSDMB with small interfering RNA in N87 and SNU‐216 cells and then cocultured them with T cells with/without IBI315. It was found that after GSDMB knockdown, IBI315‐induced pyroptotic‐like tumor cells decreased significantly (**Figure** **4**A–F). The enzyme‐linked immunosorbent assay (ELISA) analysis of coculture supernatants found that after the knockdown of GSDMB in tumor cells, the addition of IBI315 no longer caused the elevation of IL‐18 in the coculture supernatants (Figure 4G). To verify the role of GSDMB on the antitumor effect of IBI315 in vivo, GSDMB knocked‐down N87 cells (N87 sh‐GSDMB; Figure 4H) were implanted in humanized mice followed by treatment with IBI315 or IgG. The tumor inhibition rate of IBI315 on N87 was 116.8%, while the tumor inhibition effect of IBI315 was almost completely blocked after GSDMB was knocked down (Figure 4I,J). We next analyzed tumor‐infiltrated immune cells and found that knockdown of GSDMB reduced CD3^+^ and CD8^+^ T cell infiltration in tumors induced by IBI315 (Figure 4K,L). These results suggest that the tumoricidal effect of IBI315 depends on GSDMB‐cleavage‐induced pyroptosis.

### 2.5 IBI315‐Mediated Tumor Cell Pyroptosis Activates Lymphocytes and Triggers a Positive Loop of Tumor Inhibition

**Figure 5:**
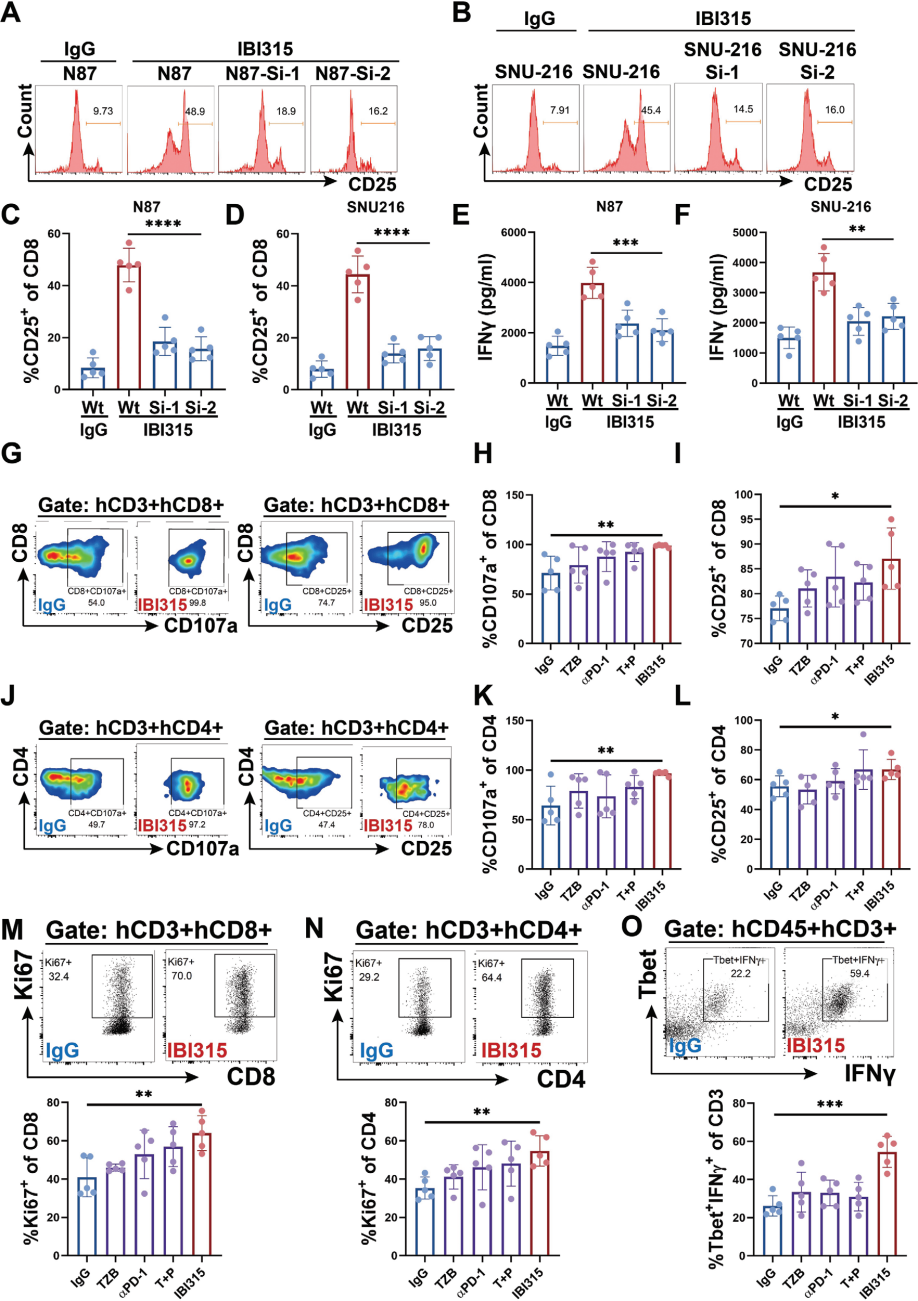
IBI315‐mediated tumor cell pyroptosis activates lymphocytes and triggers a positive loop of tumor inhibition. A–F) GSDMB expression in N87 and SNU‐216 cells was downregulated using siRNA (siGSDMB‐1 and siGSDMB‐2). The cells, with or without GSDMB knockdown (±GSDMB), were then cocultured with T cells in the presence of either IBI315 or IgG for 24 h and the supernatant was collected and used as conditioned media. Peripheral blood mononuclear cells (PBMCs) were cultured in the corresponding conditioned media for 24 h and the activation of immune cells was analyzed. A,B) Representative images and C,D) quantification of the CD25^+^ T cell population within CD8^+^ T cells of PBMCs cultured in the conditioned media from A,C) N87 (±GSDMB) or B,D) SNU‐216 (±GSDMB) cells/T cell coculture system with the indicated treatments were shown. IFNγ secretion by PBMCs treated with the conditioned media from E) the indicated N87 or F) SNU‐216 cells/T cell coculture system was measured. G–O) Human immune cell‐reconstituted NOG mice bearing PDX‐1 were subjected to treatment with IBI315, its parent antibody, or their combination (following the same administration protocol as in Figure 2). On Day 39, mice were sacrificed and tumors were collected and digested into single cells. The activation of TILs in PDX‐1 of each treatment group was analyzed by flow cytometry. Representative images and quantification of the indicated TIL populations in PDX‐1 from each group were presented. All statistical results were obtained from three independent experiments and expressed as means ± SEM. Statistical significance is indicated as *P < 0.05, **P < 0.01, ***P < 0.001, ****P < 0.0001.

**Figure 6:**
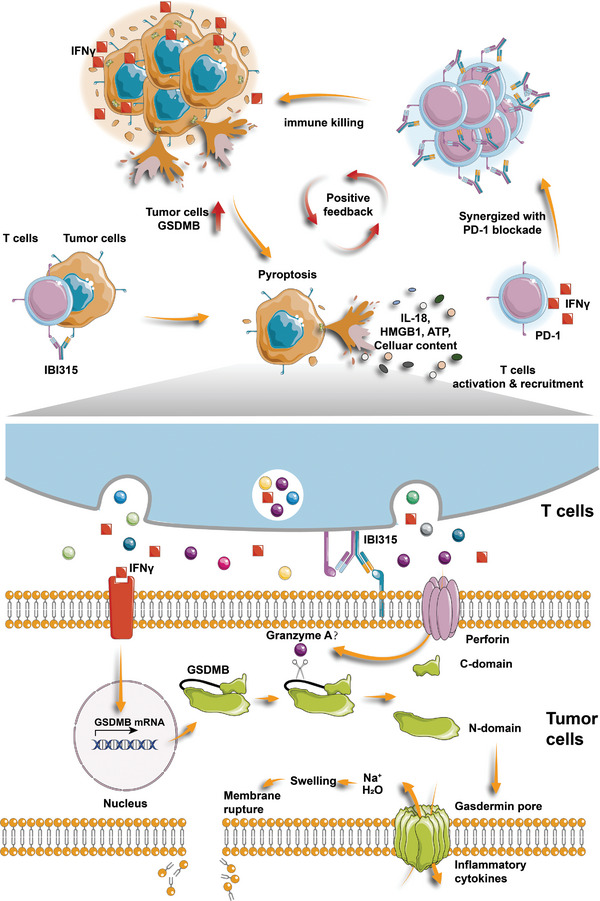
A schematic model depicting the mechanism underlying the antitumor effect of IBI315‐mediated tumor cell pyroptosis. IBI315 facilitates the formation of immune synapses between T cells and Her2‐positive tumor cells. Granzyme A might cleave GSDMB in tumor cells to generate the N‐terminal fragment, which forms Gasdermin transmembrane pores in the cell membrane and triggers pyroptosis and release of inflammatory cytokines from tumor cells. These cytokines can activate and recruit T cells and the IFNγ secreted by activated T cells can further increase the expression of GSDMB as well as PD‐L1 in tumor cells. The elevated expression of GSDMB in tumor cells enhances the occurrence of IBI315‐mediated cell pyroptosis. The blocking effect of IBI315 on PD‐1 of T cells also inhibits the interaction between PD‐1 and PD‐L1. The PD‐1 blocking function of IBI315 enhances T cells activation and induces pyroptosis in more tumor cells, thereby forming a positive feedback loop of T cell activation and tumor cell pyroptosis. All components of the mechanism are labeled in the figure for easy understanding.

To explore whether IBI315‐mediated pyroptosis could further activate T cells, we compared the activation of immune cells cultured in condition media of IgG‐treated and IBI315‐treated N87 and SNU‐216 cells/T cells cocultures, respectively. The conditioned media of IBI315‐treated groups (N87 and SNU‐216) could increase CD25 surface expression (**Figure** **5**A–D) and IFNγ secretion (Figure 5E,F) of CD8^+^ lymphocytes, which indicated enhanced CD8^+^ T cell activation. Interestingly, when we treated tumor cells with IFNγ, GSDMB was also increased in tumor cells (Figure S10A, Supporting Information). To verify IBI315‐mediated pyroptosis in T cell activation in vivo, we performed flow cytometry analysis on the intratumoral infiltrating lymphocytes of Her2‐positive gastric cancer PDX‐1 and found that compared with the control IgG group, the tumor‐infiltrating CD8^+^ and CD4^+^ lymphocytes in the IBI315 treatment group had a higher proportion of Ki67 expression (Figure 5M,N), indicating IBI315 could promote the expansion of CD8^+^ and CD4^+^ lymphocytes in tumors. Tumor‐infiltrating CD4^+^ and CD8^+^ lymphocytes exhibited a higher proportion of cells positive for CD107a, CD25, markers of lymphocyte activation (Figure 5G–L). Tbet is a transcription factor required for TH1 cell differentiation and IFNγ production.^[^ ^19^ ^]^ We found a higher proportion of Tbet^+^IFNγ^+^ cells in intratumoral CD3^+^ lymphocytes in the IBI315‐treated mice compared to the control group, the parental mAbs group or the parental two‐drug combination group (Figure 5O). These results collectively suggest that IBI315 can promote the activation of CD8^+^ and CD4^+^ lymphocytes and the secretion of IFNγ. In addition, we uncovered that compared with other groups, cytotoxic CD8^+^ lymphocytes in the IBI315‐treated group had a higher proportion of cells positive for granzyme A (GrA, Figure 3I), which revealed the enhanced killing ability of CD8^+^ lymphocytes in the IBI315‐treated group. Therefore, we inferred that IBI315 could induce tumor cell pyroptosis, and the inflammatory cytokines released by pyroptosis could further recruit and activate lymphocytes. Activated T cells often express higher levels of PD‐1, and as we have previously observed, gastric cancer cells can also induce upregulation of PD‐1 on T cells (Figure S1, Supporting Information). Moreover, MLR experiments showed that IBI315 has a PD‐1 blocking effect consistent with the parental PD‐1 antibody, activating T cells. Meanwhile, the IFNγ secreted by activated T cells leads to an increase in GSDMB as well as PD‐L1 expression on tumor cells (Figure S10, Supporting Information). The elevated expression of GSDMB in tumor cells enhances the occurrence of IBI315‐mediated cell pyroptosis. The blocking effect of IBI315 on PD‐1 of T cells also inhibits the interaction between PD‐1 and PD‐L1. Therefore, we inferred that IBI315 could further enhance T cell cytotoxicity by blocking PD‐1 on T cells, leading to increased occurrence of tumor cell pyroptosis, forming a positive feedback loop of T cell activation and tumor cell pyroptosis (**Figure** **6**).

## 3 Discussion

Herein, we found that IBI315, combined with traditional Her2‐targeted therapy and PD‐1 immune checkpoint inhibitors, yielded dual blocking activity of Her2 and PD‐1 in both in vitro and in vivo experiments. Bispecific antibody targeting both PD‐1 and Her2 could crosslink Her2‐positive tumor cells with T cells to form PD‐1 immune synapses to guide the killing of tumor cells without antigen recognition.^[^ ^11^, ^12^, ^13^, ^14^, ^15^ ^]^ We observed that IBI315 could kill target cells to a large extent in the tumor cells or organoid/T cells coculture system, whereas the killing effect was not observed in the parental mAbs or parental two‐drug combination groups. In the coculture system, T cells in the IBI315‐treated group could adhere to tumor cells. We speculate that the killing effect of IBI315 on tumor cells is related to the immune synapse it induces.

Moreover, we noticed that many tumor cells underwent pyroptosis in the tumor cells/T cells coculture system treated with IBI315. It is well‐established that pyroptosis is an inflammatory programmed cell death characterized by the continuous expansion of cells until the cell membrane ruptures, resulting in the release of cellular contents and the activation of a strong inflammatory response. Classical pyroptosis mainly relies on the activation of caspase family proteins by the inflammasome, which cleaves and activates gasdermins, and the activated gasdermins are translocated to the membrane to form gasdermin pores, resulting in cell swelling, cytoplasmic efflux, and ultimately cell membrane rupture.^[^ ^20^, ^21^ ^]^ There is a growing consensus that pyroptosis induced by tumor cell pyroptosis inducers can improve the body's antitumor immune activity and, when combined with immune checkpoint inhibitors, can transform “cold tumors” into “hot tumors,” thereby exerting a synergistic antitumor effect.^[^ ^17^, ^22^, ^23^, ^24^, ^25^ ^]^ Accordingly, the occurrence of pyroptosis depends on the production of activated gasdermins. While screening gastric cancer cell lines, PDX‐1 and PDXO‐1, we focused on GSDMB, and in subsequent knockdown experiments, we confirmed that the pyroptosis and antitumor effects of IBI315‐mediated T cells were dependent on GSDMB. Shao and co‐workers reported that granzyme A, derived from cytotoxic lymphocytes, cleaves GSDMB to generate activated GSDMB, leading to the induction of pyroptosis.^[^ ^17^ ^]^ Current evidence suggests that the N‐terminal fragment of GSDMB resulting from cleavage by Neutrophil Elastase or caspases is incapable of inducing pyroptosis, whereas cleavage by granzyme A generates an N‐terminal fragment with the ability to induce pyroptosis.^[^ ^18^ ^]^ Our findings also demonstrated that the expression of granzyme A in infiltrating immune cells exhibited changes consistent with treatment response (Figure 3H,J). Based on these observations, we hypothesized that IBI315‐induced pyroptosis in tumor cells may occur through the mediation of granzyme A secretion by cytotoxic lymphocytes, contributing to the cleavage of GSDMB. However, the available evidence in this study is insufficient to directly demonstrate that the activation of GSDMB induced by IBI315 is mediated by granzyme A. Further investigations are warranted to elucidate the underlying mechanisms of GSDMB activation. GSDMB encompasses six distinct isoforms and emerging evidence highlights the indispensability of exon 6 translation in facilitating GSDMB‐mediated pyroptosis.^[^ ^18^, ^26^, ^27^, ^28^, ^29^ ^]^ Consequently, an intriguing avenue for future research in our study pertains to elucidating the specific isoform(s) of GSDMB that IBI315 mediates, leading to the generation of the N‐terminal fragment implicated in tumor cell pyroptosis. Additionally, recent studies have implicated protein ninjurin‐1 in the membrane rupture associated with cellular pyroptosis.^[^ ^30^ ^]^ Therefore, it is important to delve deeper into the mechanisms by which the activation of GSDMB induced by IBI315 leads to cellular membrane rupture.

Liu and co‐workers constructed a bioorthogonal chemical system and induced tumor cell pyroptosis by activating gasdermins. They found that pyroptosis increased the expression of immune activation‐related genes such as CD69, granzyme A (GZMA), and granzyme B (GZMB), while it decreased the immune‐suppression‐related genes such as CSF1, vascular endothelial growth factor A (VEGFA), and CD274.^[^ ^31^ ^]^ Another study found that methotrexate‐induced pyroptosis activated macrophages and recruited neutrophils in tumors, killing tumor cells.^[^ ^32^ ^]^ Importantly, we found that pyroptosis induced by IBI315 resulted in the release of IL‐18 from tumor cells. At the same time, when we cultured T cells in the conditioned media of the IBI315 treatment group, activation and the IFNγ secretion of T cells were observed. When we knocked down GSDMB in tumor cells, we found that pyroptosis was reduced and the IL‐18 cytokines in the supernatant decreased. When T cells were cultured in the condition media of GSDMB knocked down in tumor cells, T cells could no longer be activated. Moreover, in animal experiments, we observed that N87 cells were no longer sensitive to IBI315 following GSDMB knockdown and had decreased infiltration of CD3^+^ and CD8^+^ lymphocytes in the tumor. We inferred that the inflammatory factors released by the pyroptosis induced by IBI315 could activate T cells, promote the T cell secretion of inflammatory cytokines such as IFNγ, and then recruit more immune cells in tumors. Interestingly, we found that the expression of GSDMB increased when gastric cancer cells were treated with IFNγ, consistent with Shao and co‐workers findings. The increase in GSDMB could strengthen cell pyroptosis and antitumor effects induced by IBI315, forming a positive feedback to achieve the maximum killing of tumor cells. Cellular pyroptosis triggers the release of a plethora of cytokines. Huang and co‐workers demonstrated that the pyroptotic supernatant derived from a tumor cell coculture system induced by Chimeric Antigen Receptor T (CAR‐T) cells contained adenosine triphosphate (ATP) and HMGB1, which effectively activated macrophages, leading to the secretion of IL‐1β and IL‐6.^[^ ^33^ ^]^ Similarly, HMGB1 released during melanoma cell pyroptosis was shown to activate tumor‐associated T cells and enhance infiltration of dendritic cells within the tumor microenvironment.^[^ ^34^ ^]^ Although our study did not comprehensively screen for specific cytokines responsible for T cell activation in the pyroptotic supernatant, we observed a substantial increase in IL‐18 levels following IBI315 treatment. IL‐18 is known to possess potent T cell activating properties, stimulating the production of IFNγ from both T cells and macrophages, and augmenting the cytolytic potential of natural killer(NK) cells and T cells against malignant cells.^[^ ^35^, ^36^, ^37^ ^]^ Thus, we hypothesize that IL‐18 present in the supernatant may serve as a pivotal factor in activating T cells. In the future, it is necessary to further determine the specific cytokines for activating T cells.

It is worth noting that IFNγ is also a cytokine documented to upregulate the expression of PD‐L1 in tumor cells.^[^ ^38^ ^]^ Herein, we observed that IFNγ increased the expression of GSDMB and PD‐L1, suggesting that the combination of pyroptosis inducers and immune checkpoint inhibitors may be able to maximize the effect of killing tumor cells.

Our research found that 66% of Her2‐positive gastric cancers in the TCGA database^[^ ^39^ ^]^ harbored GSDMB amplifications. The GSDMB gene and ERBB2 gene are located at the ERBB2/NEU locus (17q12‐21), and the amplification of ERBB2 leads to the amplification of GSDMB (Figure S11, Supporting Information).^[^ ^40^, ^41^, ^42^ ^]^ Indeed, this is tantamount to installing a “bomb” for tumor cells; IBI315 bridges tumor cells and T cells and acts as the “fuse” that detonates the bomb, which induces pyroptosis of tumor cells and further activates and recruits more immune cells, causing a local inflammatory response. Therefore, IBI315 holds great potential for extensive application in Her2‐positive gastric cancer.

In conclusion, This study lends support to the potential of IBI315 as a promising bispecific antibody‐based immunotherapeutic approach in preclinical assessments, thereby expanding the therapeutic options available for patients with Her2‐positive gastric cancer.

At the same time, in the existing immunotherapy cohorts analysis, we found that GSDMB also has significant value as a biomarker to predict the efficacy of immunotherapy, yielding better performance than microsatellite instability (MSI), PD‐L1, tumor mutational burden (TMB), and other classic prediction indicators (Figure S12–S14, Supporting Information). Currently, there are no ideal and effective biomarkers for gastric cancer immunotherapy; microsatellite unstable (MSI‐H) and high tumor mutation burden in gastric cancers suggest that patients may benefit from immunotherapy.^[^ ^43^ ^]^ Given that Her2‐positive gastric cancers have a low mutation burden,^[^ ^44^ ^]^ immunotherapy is not recommended as first‐line treatment for this patient population in the existing guidelines. However, the results of KEYNOTE‐811 showed that immunotherapy, combined with Trastuzumab, and chemotherapy, greatly improved the treatment efficacy of Her2‐positive gastric cancer patients.^[^ ^7^ ^]^ MSI analysis of gastric cancer in the benefited population revealed only three patients (0.7%) were MSI‐H type gastric cancer, which may suggest that in immunotherapy, pyroptosis‐related markers should be considered in addition to considering traditional biomarkers such as MSI, TMB, and PD‐L1. Significant GSDMB amplification is present in Her2‐positive gastric cancer; although its mutation load is low, this patient population may also benefit from immunotherapy, which has been substantiated by the KEYNOTE‐811 study.^[^ ^7^ ^]^

## 4 Experimental Section

### Cell Lines, Culture Conditions, and Transfection

The N87, SNU‐216, and BT‐474 cell lines were cultured in royal park memorial institute (RPMI） medium (Hyclone) supplemented with 10% fetal bovine serum (FBS, Gibco) and maintained at 37 °C with 5% CO_2_. The SK‐BR‐3 cell line were culture in design,manufacture and engineering management (DMEM) (Hyclone) supplemented with 10% FBS. Human PBMCs (P121080605C and LP220727010) were obtained from ALLCELLS (Shanghai, China) and cultured in RPMI medium with 10% heat‐inactivated FBS.

For siRNA knockdown experiments, polyplus transfection kit (Lot# PT‐114‐15) was used to transfect N87 cells with siRNAs targeting GSDMB. Knockdown efficiency was validated by immunoblotting. The siRNA sequences used were siGSDMB‐1 (5’‐GCCUUGUUGAUGCUGAUAGAUTT‐3’) and siGSDMB‐2 (5’‐GCUGUAUGUUGUUGUCUCUAUTT‐3’).

For stable knockdown of GSDMB, lentivirus containing the PLK0.1‐PURO plasmid loaded with shGSDMB sequence (5’‐GCCUUGUUGAUGCUGAUAGAUTT‐3’) was obtained from Genechem (Shanghai, China). N87 cells were infected with the lentivirus and stable knockdown of GSDMB was selected by culturing cells in medium containing puromycin.

### SPR

The SPR method was used to measure the binding affinity of IBI315, trastuzumab, and sintilimab to human Her2 protein and human PD‐1 protein. Briefly, the ligands (Her2 or PD‐1) were immobilized onto a sensor chip surface and the analyte antibodies (IBI315, Trastuzumab, and sintilimab) were injected over the surface. The binding affinity was measured by monitoring the change in refractive index caused by the interaction between the antibodies and the ligands. The SPR data were analyzed to determine the kinetic constants, including the association rate constant (*k*_on_), the dissociation rate constant (*k*_off_), and the equilibrium dissociation constant (*K*_D_) of the interaction between the antibodies and the proteins. The experiments were performed using a Biacore T200 instrument and the data were analyzed using the Biacore T200 Evaluation software.

### MLR

The CD4^+^ cells were isolated from the PBMC using the Human CD4^+^ T cell Enrichment Kit (19052, Stemcell) according to the manufacturer's instructions. The isolated cells were then activated by adding Dynabeads human T‐Activator CD3/CD28 in a 1:1 ratio and incubated for 3 d. The appropriate antibodies (IBI315, anti‐PD‐1 antibody (sintilimab), trastuzumab, sintilimab in combination with trastuzumab) were diluted in a gradient and added to the cells. Mature dendritic cells (DCs) (Allcells, FPB‐DC002F‐C) were mixed with CD4^+^ cells at a ratio of 1:10, and the cells were cultured in the presence of the antibodies for 3 d. The concentrations of secreted IFNγ and IL‐2 in the supernatant were measured by ELISA. The half‐maximal effective concentration (EC_50_) was calculated using a three‐parameter logistic curve fit of the log agonist concentration versus the response in GraphPad Prism 8.3.0.

### Enzyme‐Linked Immunosorbent Assay

ELISA kit used for the experiment included Human IL‐18 (Dakewei, 1121802), Human IL‐2 (Dakewei, 1110203), and Human IFNγ (Dakewei, 1110002). The procedure was carried out according to the instructions provided with the kit. Washing buffer (50×) and ready‐to‐use solutions were taken out of the kit 20 min prior to use and allowed to equilibrate to room temperature. Samples (including standards) and blanks were prepared in triplicate. Cytokine standard (diluted) was added to the standard wells, sample was added to the sample wells, and Dilution buffer R (1×) was added to the blank wells. Biotinylated antibody working solution was added to each well and incubated for 2 h at room temperature. Plates were washed three times with washing buffer, Streptavidin‐Horseradish peroxidase (HRP) working solution was added to each well, and plates were incubated for 20 min at room temperature. Plates were washed again, TMB was added to each well and incubated for 5–30 min at room temperature in the dark. The reaction was stopped by adding stop solution to each well and absorbance was measured at 450 nm using a microplate reader (MolecuLar Device Inc.).

### Cytokine Release Syndrome Determination

The secretion levels of IL‐2, IL‐4, IL‐6, IL‐10, TNFα, and IL‐17A were assessed using the BD Cytometric Bead Array (560484, BD Biosciences). To perform the assay, the anti‐PD‐1 antibody and IBI315 were serially diluted in PBMC culture medium to obtain ten concentrations, with a maximum concentration of 1530 nm (2×) and a fivefold dilution factor. The diluted antibodies were added to individual wells of a 96‐well plate (100 µL per well), followed by the addition of 1×10^5^ PBMCs (LP220727010) per well (100 µL), resulting in a total volume of 200 µL per well. After incubating for 24 and 48 h, the supernatants were collected and the BD Cytometric Bead Array protocol was then followed. Briefly, the capture beads provided in the kit were incubated with the indicated supernatants to allow for specific cytokine binding. Pectinesterase (PE) detection reagents were subsequently added to form bead‐cytokine‐PE complexes. Following bead washing, flow cytometry was employed to acquire data on the complexes. The acquired data were analyzed using GraphPad Prism 8.3.0, and the secretion levels of IL‐2, IL‐4, IL‐6, IL‐10, TNFα, and IL‐17A were determined based on fluorescence intensity relative to a standard curve.

### PDX and Organoid Establishment

Tumor samples were obtained from patients treated at the First Affiliated Hospital of Zhejiang University (Hangzhou, China). Informed written consent was provided by all patients and the investigations were conducted in compliance with the study protocol approved by the Clinical Research Ethics Committee of the First Affiliated Hospital of Zhejiang University (reference number: 2022344). PDXs were generated by subcutaneously implanting tumor tissue into 4‐week‐old non obese diabetes‐server combined immune deficiency (NOD‐SCID) mice (Vita River, China). All procedures were performed in accordance with institutionally approved institutional animal care and use committee (IACUC) protocols.

To generate organoids, shredded PDX tissue was dissociated into single cells using gentle cell dissociation reagent (07174, STEMCELL) according to the manufacturer's instructions. The cells were then suspended in organoid basal medium, which consisted of advanced DMEM/F12 medium containing 15 mmol L^−1^ Hepes, and filtered through a 70 µm cell strainer (Falcon). The cells were resuspended in 2 mL of organoid basal medium and the cell concentration was determined using a hemocytometer. The cells were then resuspended in Matrigel (356231, Corning) at a concentration of 1.6×10^5^ mL^−1^, and 50 µL of the organoid‐Matrigel suspension was slowly added to the center of a 24‐well plate prewarmed to 37 °C. After solidification at 37 °C for 20 min, IntestiCult Organoid Growth Medium (Human, 06010, STEMCELL) containing 10 µm Y‐27632 dihydrochloride (Y0503, Sigma) was added, and the organoids were incubated at 37 °C and 5% CO_2_.

### Establishment Tumor Cells or Organoids/T Cells Coculture Cytotoxicity System

The coculture system was established as previously described and the schematic diagram of the system was shown in Figure S2.^[^ ^16^ ^]^ Briefly, tumor cells or organoids were cocultured with activated T cells isolated from PBMCs using the Pan T cell isolation kit (130‐096‐535, Miltenyi) and indicated antibodies (600 nm) in 96‐well plates for 24 h, with an effector‐to‐target ratio (E:T) of 10:1. Subsequently, the cytotoxicity of T cells was measured by lactate dehydrogenase (LDH) release with the CytoTox 96 nonradioactive cytotoxicity assay (G1780, Promega) according to the manufacturer's protocol. Briefly, quadruplicate wells were set up to measure absorbance values at 490 nm, which represent LDH release from effector cells, target cells, experimental wells, and background values for volume correction control, culture medium background control, and LDH positive control. The average absorbance value for the culture medium background was subtracted from all absorbance values for experimental, target cell spontaneous LDH release, and effector cell spontaneous LDH release to calculate percent cytotoxicity. The average absorbance values were also subtracted for the volume correction control from the absorbance values obtained for the target cell maximum LDH release control. The corrected values obtained were used in the following formula.

(1) $$\begin{array}{ccc} & & \%\mathrm{Cytotoxixity}\\ & & \;=\frac{\mathrm{Experimental}-\mathrm{Effector}\;\mathrm{spontaneous}-\mathrm{Target}\;\mathrm{spontaneous}}{\mathrm{Target}\;\mathrm{maximum}-\mathrm{Target}\;\mathrm{spontaneous}}\times 100 \end{array}$$

The levels of IL‐18 in the supernatants were also measured.

To observe the antibody‐mediated killing effect of PBMCs, PBMCs were used instead of T cells.

### Quantification of Pyroptotic Cells

N87 or SNU‐216 cells were digested and washed with phosphate buffer saline (PBS) to remove excess FBS, and the cell concentration was adjusted to 10^6^ mL^−1^. A certain volume of cell suspension was taken according to the experimental requirements, and Carboxyfluorescein succinimidyl ester (CFSE) stock solution (C34554, Invitrogen) was prepared according to the instructions and added to the cell suspension at a 1:2000 ratio. The mixture was then incubated for 20 min at 37 °C in the dark. After staining, five times the volume of the cell suspension of culture medium (containing 10% FBS) was added to neutralize excess staining solution at 37 °C. The stained cells were then washed twice with PBS, centrifuged at 400 g for 5 min, and the supernatant was discarded. The resulting cell suspension was resuspended in cell culture medium, counted, and cocultured with T cells at an effector‐to‐target ratio of 10:1 after activation. The corresponding antibodies were added according to the experimental design and the cells were cocultured for 6 h. The pyroptotic cells were identified by characteristic membrane bubbling and swelling under a fluorescence microscope. Each treatment group was randomly photographed with five images, and the proportion of pyroptotic cells in each image was quantified.

### ADCC Assay

NK cells were isolated from PBMCs using the NK cells isolation kit (130‐092‐657, Miltenyi) according to the manufacturer's instructions, and the concentration was adjusted to 5×10^5^ mL^−1^. N87 cells were digested with trypsin and the cell concentration was adjusted to 1×10^5^ mL^−1^. 50 µL of the NK cell suspension and 50 µL of the N87 cell suspension were added to each well of a 96‐well flat‐bottom plate and mixed evenly to achieve an effector‐to‐target ratio (E:T) of 5:1. The concentration of the required antibody solution was adjusted and gradient diluted. 50 µL of each diluted antibody was added to the corresponding coculture system in the 96‐well plate, and the plate was centrifuged at 250 g for 4 min to ensure sufficient contact between the effector cells and target cells. The plate was then incubated for 4 h at 37 °C with 5% CO_2_ in a cell culture incubator. The level of LDH in the supernatant was measured using the CytoTox 96 nonradioactive cytotoxicity assay kit to calculate the cytotoxicity of each group, as described above.

### Cell Bridge Assay

Activated T cells were labeled with Cell Trace CFSE, and N87 cells were labeled with Cell Trace Deep Red for 20 min at 37 °C, respectively. The cell concentrations were adjusted to 2×10^5^ mL^−1^ and the antibodies (IBI315, sintilimab and trastuzumab) were diluted to a concentration of 600 nm. Next, 50 µL of the activated T cell suspension and the N87 cell suspension were mixed with the corresponding diluted antibodies in a U‐bottom 96‐well plate and incubated at 4 °C for 30 min. Following this, the cell pellets were washed twice with PBS and analyzed by flow cytometry.

### Animal Studies

Four‐week‐old female NOD/ShiLtj‐scid IL2rg<null> (NOG) mice were purchased from Vita River (Beijing, China). All procedures were performed in accordance with institutionally approved IACUC protocols and approved by the Animal Experimental Ethical Inspection of the First Affiliated Hospital, Zhejiang University School of Medicine with the reference number of 20221594. Mice were subcutaneously injected in the right flank with N87 cells (2×10^6^) or implanted with PDX‐1 (2 mm × 2 mm × 2 mm) in day 0, and then were tail vein injected with 2.5×10^6^ PBMCs (in day 3 for N87 cells, day 7 for PDX‐1). Mice with greater than 20% of CD45^+^ cells in peripheral blood and tumor size in the range of 80–100 mm^3^ were randomly divided into five groups (six mice/treatment group for N87 tumor and seven mice/treatment group for PDX‐1) with comparable average tumor size and administered intraperitoneally every 3 d for 2 weeks with: IBI315 (5 mg kg^−1^), IgG isotype control antibody (5 mg kg^−1^), trastuzumab (5 mg kg^−1^), sintilimab (5 mg kg^−1^) or the combination trastuzumab (5 mg kg^−1^) and sintilimab (5 mg kg^−1^). Tumors were measured every 3 d (1/2*ab* ^2^, where *a* represents long diameter and *b* represents short diameter of tumor). The formula used to calculate the relative tumor growth inhibition (TGI) is as follows: TGI (%) = 100% x (Tvolcontrol – Tvoltreated) / (Tvolcontrol – Tvolpredose). In this formula, Tvolcontrol – Tvoltreated represents the difference between the tumor volume at the end of the study in the control group and the treated group, while Tvolcontrol – Tvolpredose represents the difference between the tumor volume at the end of the study and the volume before treatment in the control group. Mice were euthanized if they showed signs of discomfort or if the total tumor volume exceeded 2500 mm^3^. After 1–2 weeks of drug withdrawal, mice were euthanized, and tumor tissues were collected for further immunohistochemistry (IHC) and flow cytometry analysis.

### Tumor Single Cell Suspension Preparation

Tumors were digested with a lysis solution containing RPMI medium, Liberase research grade enzyme blend (Liberase DL) (0.12 mg mL^−1^), and DNase I (20 U mL^−1^) on a shaker at 37 °C for 30 min. The resulting cell suspension and tissues were then ground and filtered through a 40‐µm cell strainer (Falcon) and the flow‐through was washed with PBS three times. Afterward, red blood cells were lysed in the cell pellet with a red blood cell lysis buffer (00‐4333‐57, Invitrogen) according to the manufacturer's protocol. Finally, cells were suspended in an appropriate amount of PBS containing 2% FBS for flow analysis.

### TIDE Database

The Tumor Immune Dysfunction and Exclusion (TIDE) database (http://tide.dfci.harvard.edu/) is a computational framework developed to evaluate the potential of tumor immune escape from the gene expression profiles of cancer samples. It includes data from clinical immunotherapy cohorts containing existing transcriptome data, and the included databases can be analyzed to obtain a module for comparing custom biomarkers with other published biomarkers based on their predictive power for response outcome and overall survival.^[^ ^45^, ^46^ ^]^ Here, TIDE was used to analyze the predictive value of GSDMB for immunotherapy.

### Statistical Analysis

All data were presented as mean ± standard error of the mean (SEM). An unpaired two‐tailed Student's *t*‐test or nonparametric test was performed for the comparison of two groups. For comparisons of more than two groups, one‐way analysis of variance was conducted. A *p*‐value less than 0.05 was considered statistically significant.

### Additional Information

The antibodies and reagents used in this study are listed in Table S1 in the Supporting Information.

## Conflict of Interest

M.Z., J.X., W.W., and B.C. are employees of Innovent Biologics (Suzhou). All other authors declare no potential conflicts of interest.

## Author Contributions

W.L., Y.Z., and Y.Y. contributed equally to this work. The experimental plan was designed by W.L., Y.Z., Y.Y., L.T., and Q.D.Z. B.L., M.Z., J.X., Y.C., W.W., B.C., W.L., X.C., J.Liu, H.H.W., and F.T. performed experiments and analyzed data. X.Y. and H.Y.W. conducted the revised experiment. W.L. and Q.D.Z. wrote the paper and it was revised by J.Lu and all other authors.

## Supporting information

Supporting Information

## Data Availability

Research data are not shared.
