# Supplementary material for: Anti‐PD‐1/Her2 Bispecific Antibody IBI315 Enhances the Treatment Effect of Her2‐Positive Gastric Cancer through Gasdermin B‐Cleavage Induced Pyroptosis
Source: Adv Sci (Weinh). 2023 Aug 16;10(30):2303908. doi: 10.1002/advs.202303908 (PMC10602533; doi:10.1002/advs.202303908)
Supplement: Supplementary file 1 — Supporting Information [file ADVS-10-2303908-s001.pdf]

ADVANCED SCIENCE
Open Access

Supporting Information

for *Adv. Sci.*, DOI 10.1002/advs.202303908

Anti-PD-1/Her2 Bispecific Antibody IBI315 Enhances the Treatment Effect of Her2-Positive Gastric Cancer through Gasdermin B-Cleavage Induced Pyroptosis

Wu Lin, Yingzi Zhang, Yan Yang, Ben Lin, Mengjia Zhu, Jinling Xu, YiRan Chen, Weiwei Wu, Bingliang Chen, Xiangliu Chen, Jin Liu, Haohao Wang, Fei Teng, Xiongfei Yu, Haiyong Wang, Jun Lu, Quan Zhou and Lisong Teng**

Supporting Information

Title

Anti-PD-1/Her2 bispecific antibody IBI315 enhances the treatment effect of Her2-positive gastric cancer through gasdermin B-cleavage induced pyroptosis

Wu Lin†, Yingzi Zhang†, Yan Yang†, Ben Lin, Mengjia Zhu, Jinling Xu, YiRan Chen, Weiwei Wu, Bingliang Chen, Xiangliu Chen, Jin Liu, Haohao Wang, Fei Teng, Xiongfei Yu, Haiyong Wang, Jun Lu, Quan D Zhou, Lisong Teng**

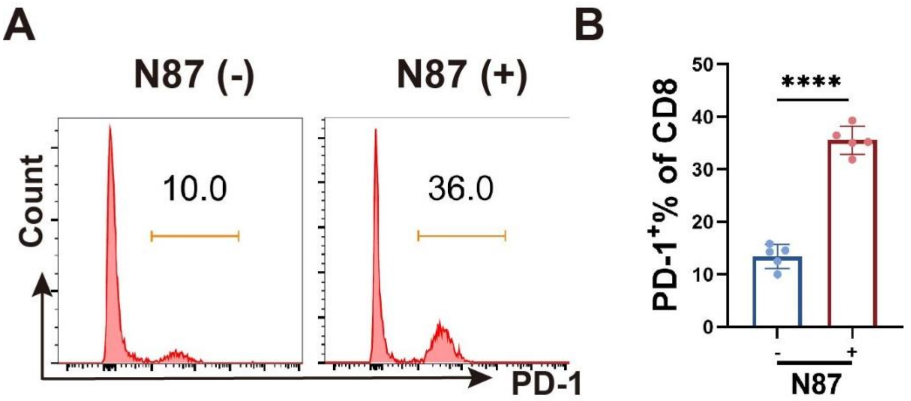

Figure S1 Elevated PD-1 expression on T cells following co-culture with gastric cancer cells. After co-culture with N87 cells or alone for 3 days, $CD8^+$ T cells were analyzed for the proportion of $PD\text{-}1^+$ cells. A) Representative flow cytometry plots, B) statistical analysis of the data. Statistical significance is indicated as ****P<0.0001.

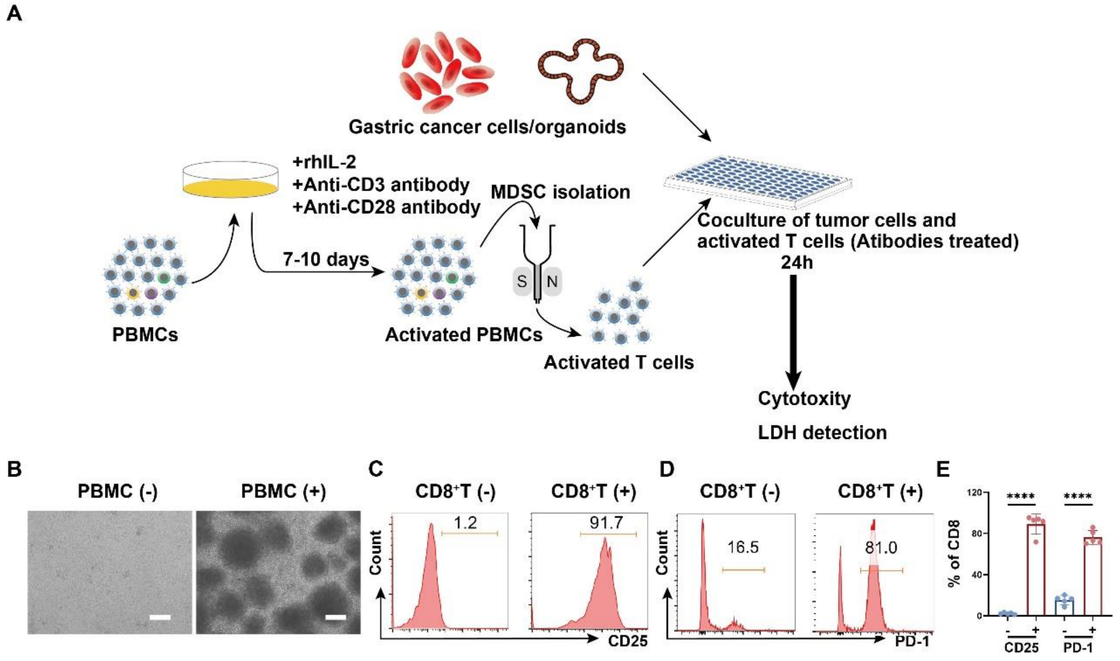

Figure S2 Establishment tumor cells or organoids/T cells co-culture cytotoxicity system. A) Schematic diagram of the system. PBMCs were activated with anti-CD3 antibody, anti-CD28

antibody, and IL-2 for 7-10 days, and T cells were subsequently enriched using magnetic beads. T cells were then co-cultured with gastric cancer cells or organoids at an effector-to-target ratio of 10:1, with the addition of indicated antibodies. After 24 hours, the supernatant was collected to measure the level of LDH, and the cytotoxicity of each group was compared (detailed calculation methods are described in the experimental section). B) Morphological changes of PBMCs after activation were observed under a microscope. C-E) Representative flow cytometry plots and statistical data (E) showing CD25 (C) and PD-1 (D) expression in CD8-positive lymphocytes. Scale bar: 100 µm. Statistical significance is indicated as ****P<0.0001.

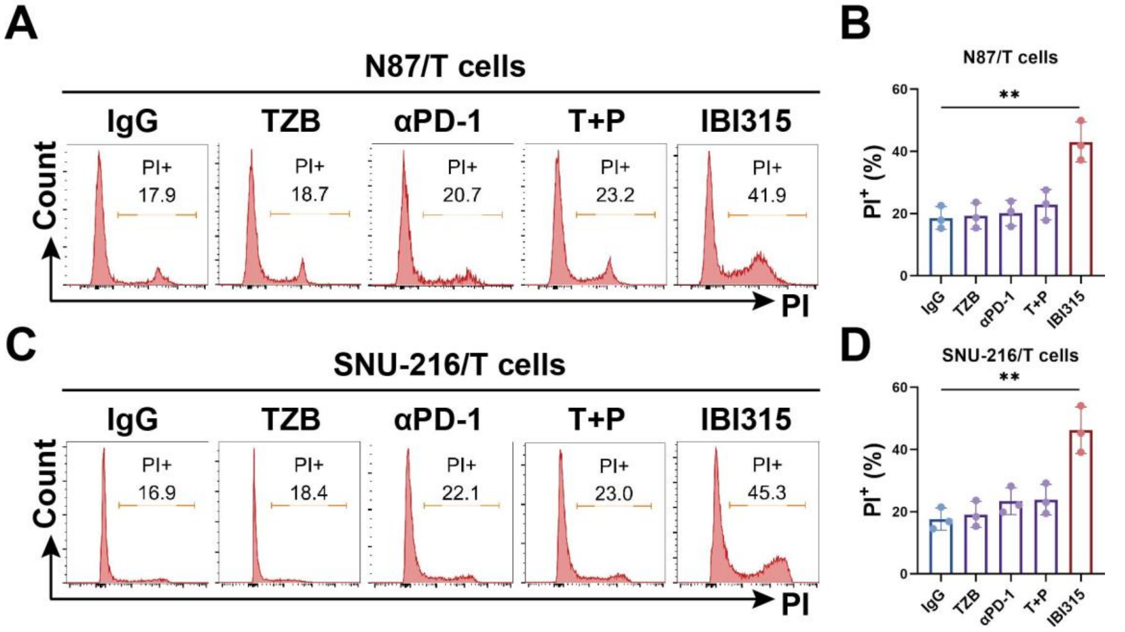

Figure S3 IBI315 mediates T cell-mediated cytotoxicity against Her2-positive gastric cancer cells. A-D) Her2-positive gastric cancer cells (N87 and SNU-216) were pre-labeled with CFSE and treated with IBI315, its parent antibody, or their combination for 24 hours in the presence of T cells. Representative images and quantification of tumor cell death were analyzed by flow cytometry, and the percentage of PI^+ tumor cells ($PI^+/CFSE^+$) were measured in N87/T cells (A, B) and SNU-216/T cells (C, D) co-culture systems.Statistical significance is indicated as **P<0.01.

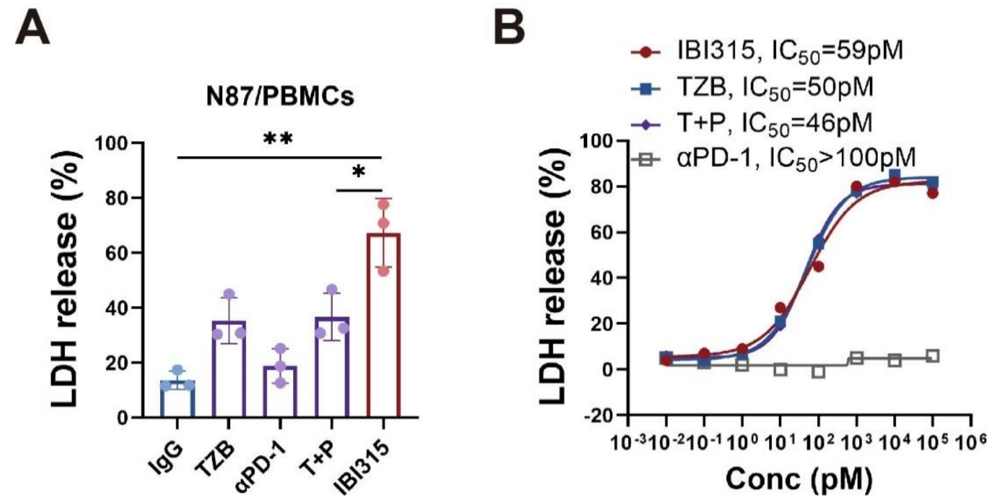

Figure S4 IBI315 retains the ADCC activity of its parental trastuzumab. A-B) N87 cells were treated with IBI315, its parent antibody, or their combination in the presence of PBMCs or NK cells. Quantification of tumor cell death by measuring LDH release as indicated treatment of N87/PBMCs co-culture system is presented (A). Comparison of NK cell-mediated cytotoxicity of N87 cells by indicated treatment (B). Statistical significance is indicated as *P<0.05 and **P<0.01.

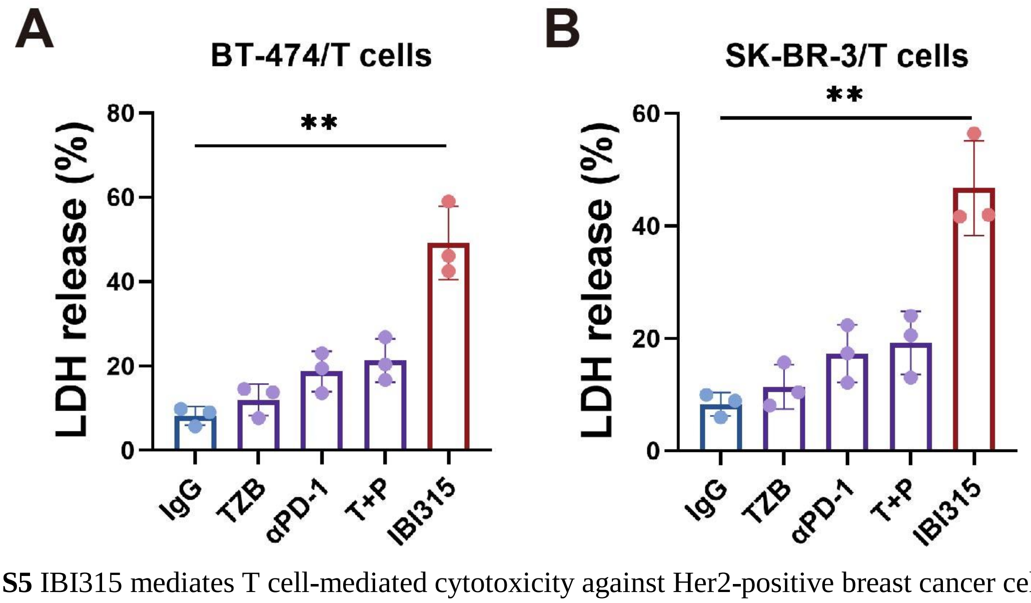

Figure S5 IBI315 mediates T cell-mediated cytotoxicity against Her2-positive breast cancer cells. A-B) Her2-positive breast cancer cells (BT-474 and SK-BR-3) were treated with IBI315, its parent antibody, or their combination for 24 hours in the presence of T cells. Quantification of tumor cell death by measuring LDH release as indicated treatment of BT-474/T cells (A) and SK-BR-3/T cells (B) co-culture system is presented. Statistical significance is indicated as **P<0.01.

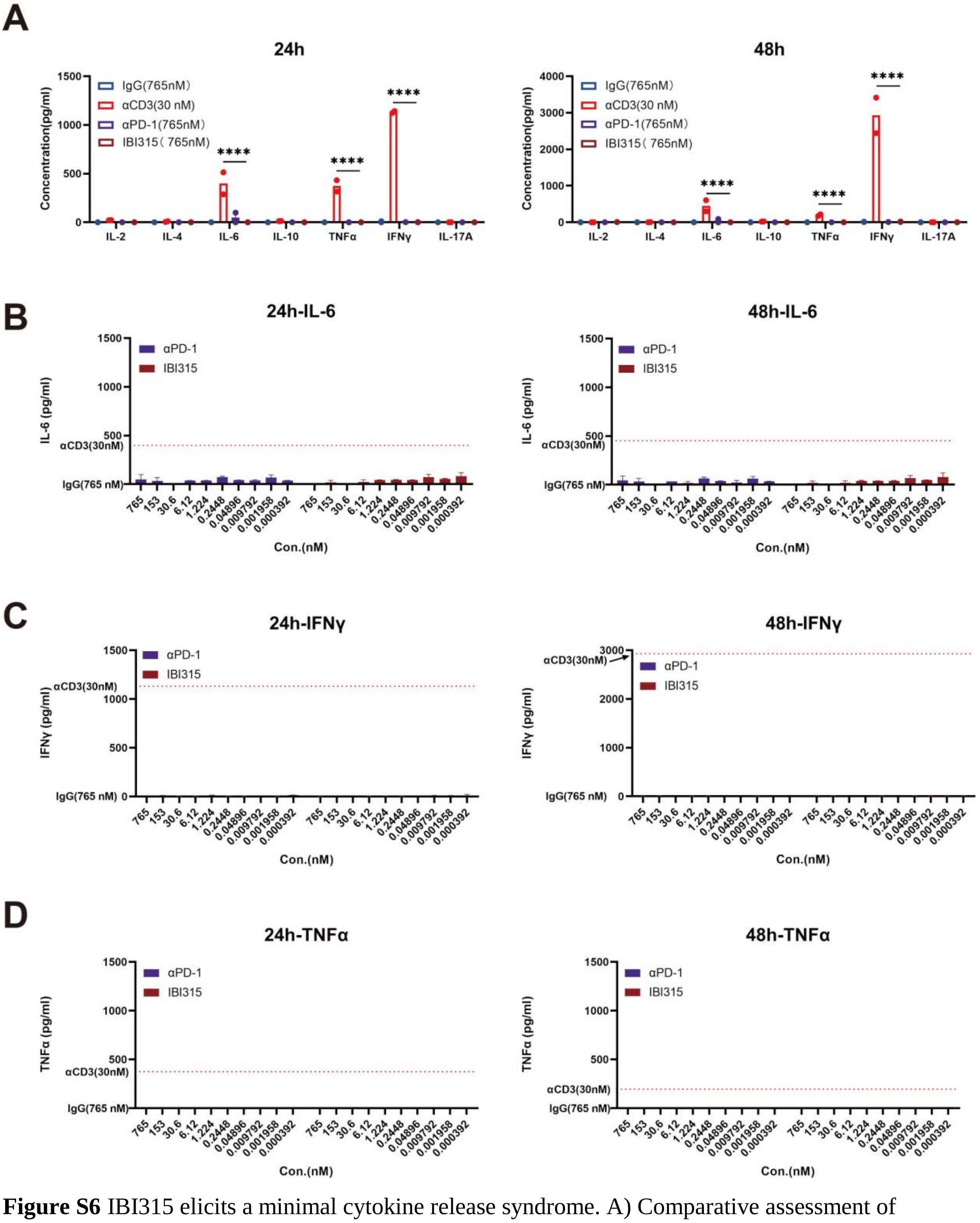

Figure S6 IBI315 elicits a minimal cytokine release syndrome. A) Comparative assessment of cytokine levels in the supernatant of human PBMCs treated with IgG (765nM), anti-CD3 antibody (positive control, 30nM), anti-PD-1 antibody (765nM), and IBI315 (765nM) for 24 and 48 hours. B-D) Serial dilutions of anti-PD-1 antibody and IBI315 were applied to human PBMCs for 24 and 48 hours, and the supernatant was analyzed for IL-6 (B), IFNγ (C), and TNFα (D) levels. The red dashed line represents the average cytokine concentration in the supernatant after treatment with 30nM anti-CD3 antibody (positive control) under corresponding conditions. The average cytokine concentration in the supernatant after treatment with 765nM IgG under corresponding conditions are overlapping with the x-axis. Statistical significance is indicated as ****P<0.0001.

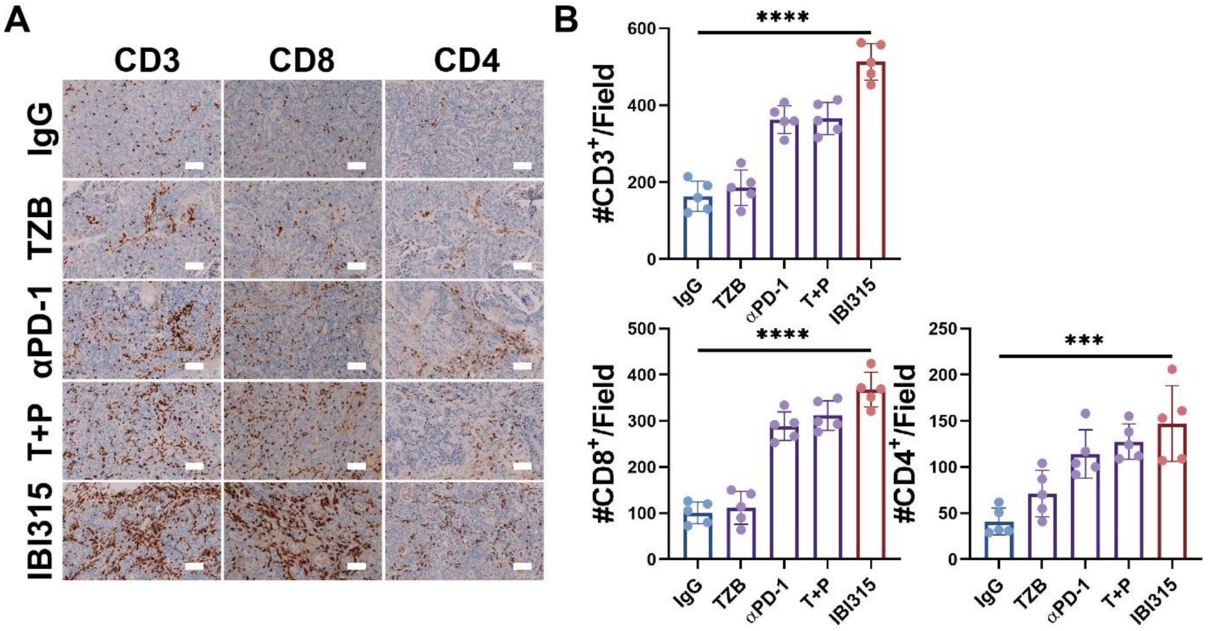

Figure S7 IBI315 enhanced immune cell infiltration in N87 tumors. A-B) Human immune cell-reconstituted NOG mice bearing N87 tumors were subjected to treatment with IBI315, its parent antibody, or their combination. Representative images of $CD3^+$, $CD8^+$, and $CD4^+$ T cell infiltration in N87 tumors of each group (A). Quantification results of immune cell infiltration (B). All statistical results were obtained from three independent experiments and expressed as means ± SEM. Abbreviations: TZB: trastuzumab, αPD1: anti-PD1 antibody (sintilimab), T+P: trastuzumab + sintilimab. Scale bar = 50 µm. *P<0.05, **P<0.01, ***P<0.001, ****P<0.0001.

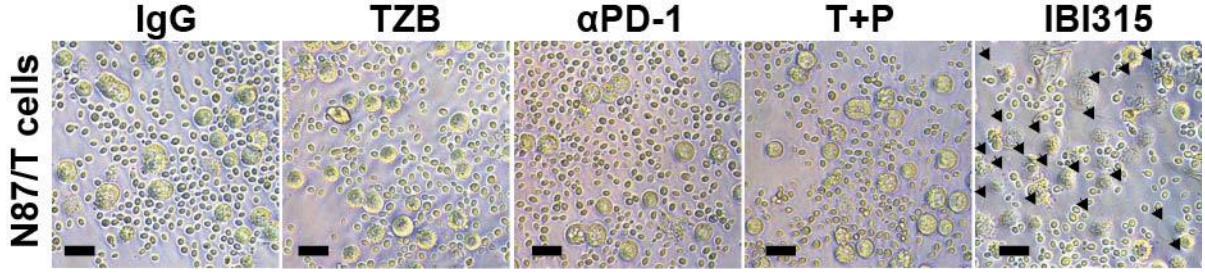

Figure S8 IBI315 induces pyroptosis-like morphological changes in N87 cells in the N87/T cells co-culture system. N87 cells were treated with IBI315, its parent antibody, or their combination for 6 hours in the presence of T cells, and images were captured. Pyroptotic cells are marked with black arrows. Scale bar: 100 µm. Abbreviations: TZB: trastuzumab, αPD1: anti-PD1 antibody (sintilimab), T+P: trastuzumab+sintilimab.

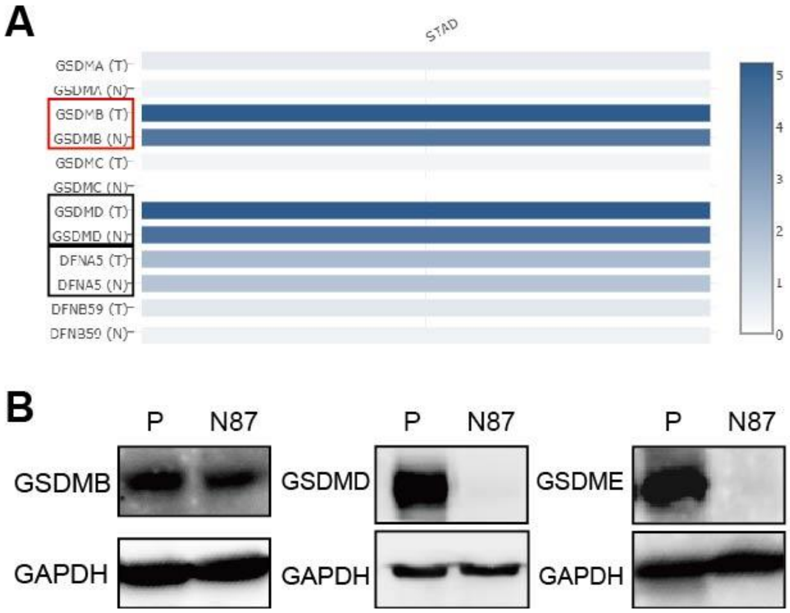

Figure S9 Expression of GSDM family in gastric cancer. A) Relative expression levels of GSDM family members (DFNA5 refers to GSDME) in gastric cancer and paracancer tissues from the TCGA database. B) Immunoblotting analysis of the expression of GSDMB, GSDMD and GSDME in N87 cells, with positive control represented by gastric cancer tissues (P).

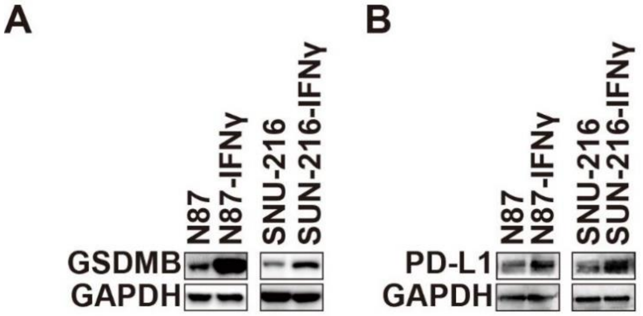

Figure S10 IFNγ primes GSDMB and PD-L1 expression. A-B) Immunoblotting of GSDMB (A) and PD-L1 (B) expression in N87 or SNU-216 cells treated with IFNγ (100ng/mL) for 24 hours.

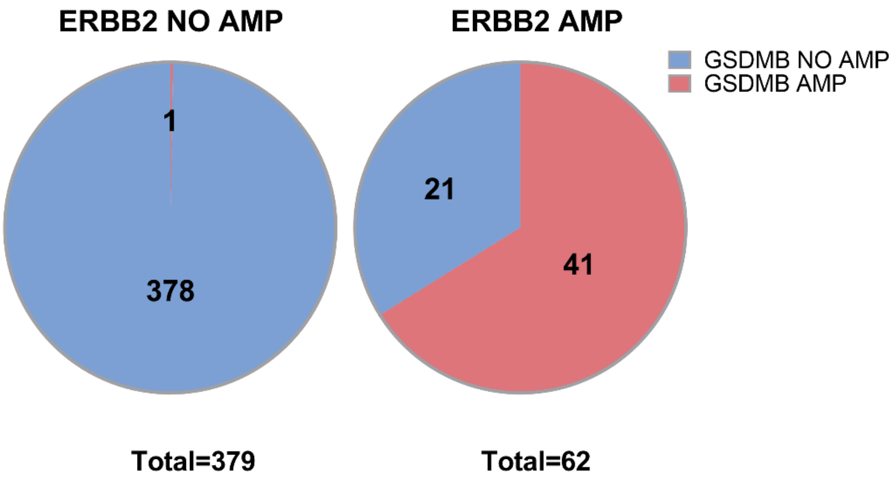

Figure S11 Proportion of GSDMB amplification in ERBB2-amplified gastric cancer. Analysis of the TCGA database (n=441) revealed a higher frequency of GSDMB amplification in ERBB2-amplified gastric cancers.

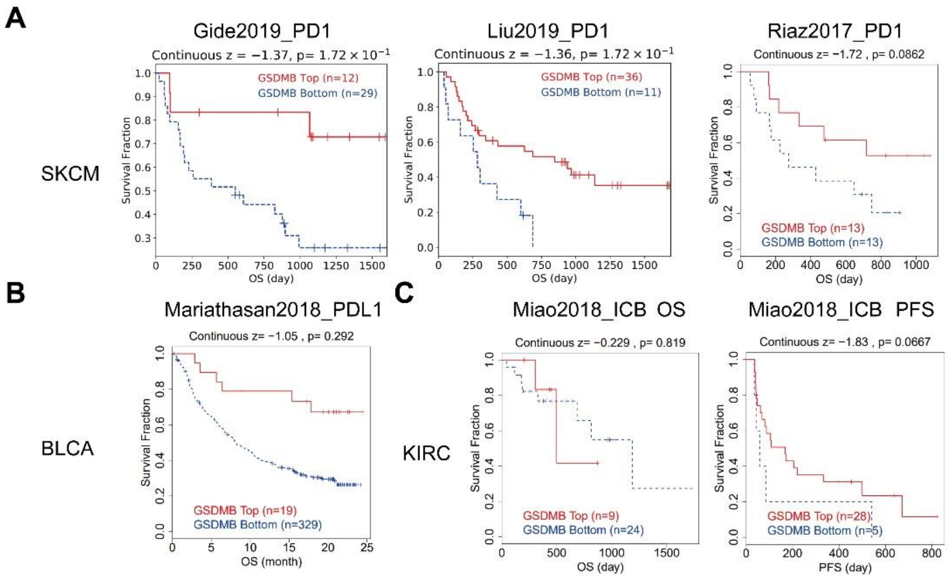

Figure S12 Association of GSDMB expression with improved prognosis in immunotherapy. A-C) TIDE database analysis of the correlation between GSDMB expression and treatment outcomes in melanoma (A), bladder cancer (B), and kidney cancer (C) immunotherapy cohorts.

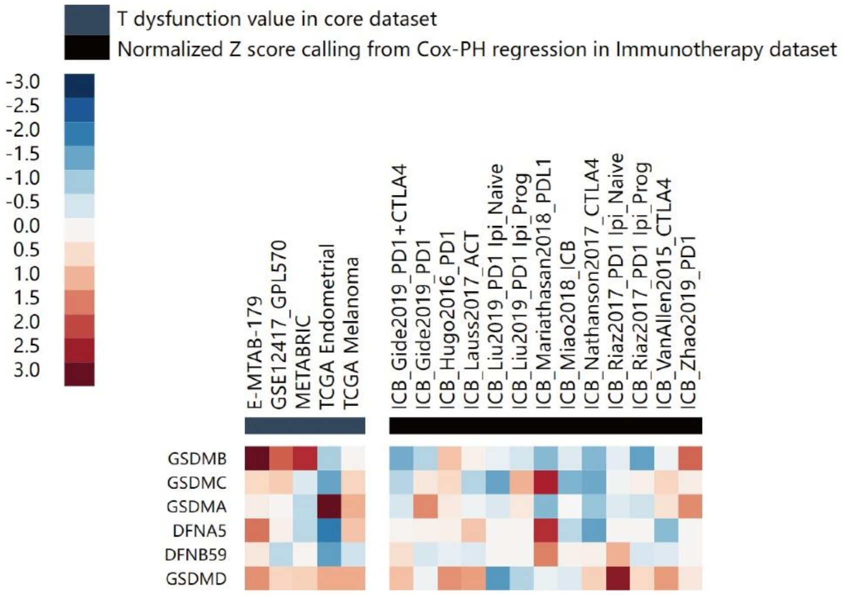

Figure S13 Analysis of GSDMB expression revealed an association with altered T cell function and increased responsiveness to immunotherapy. Using the TIDE database, we evaluated the expression of GSDMs family members and their association with treatment outcomes in various immunotherapy cohorts.

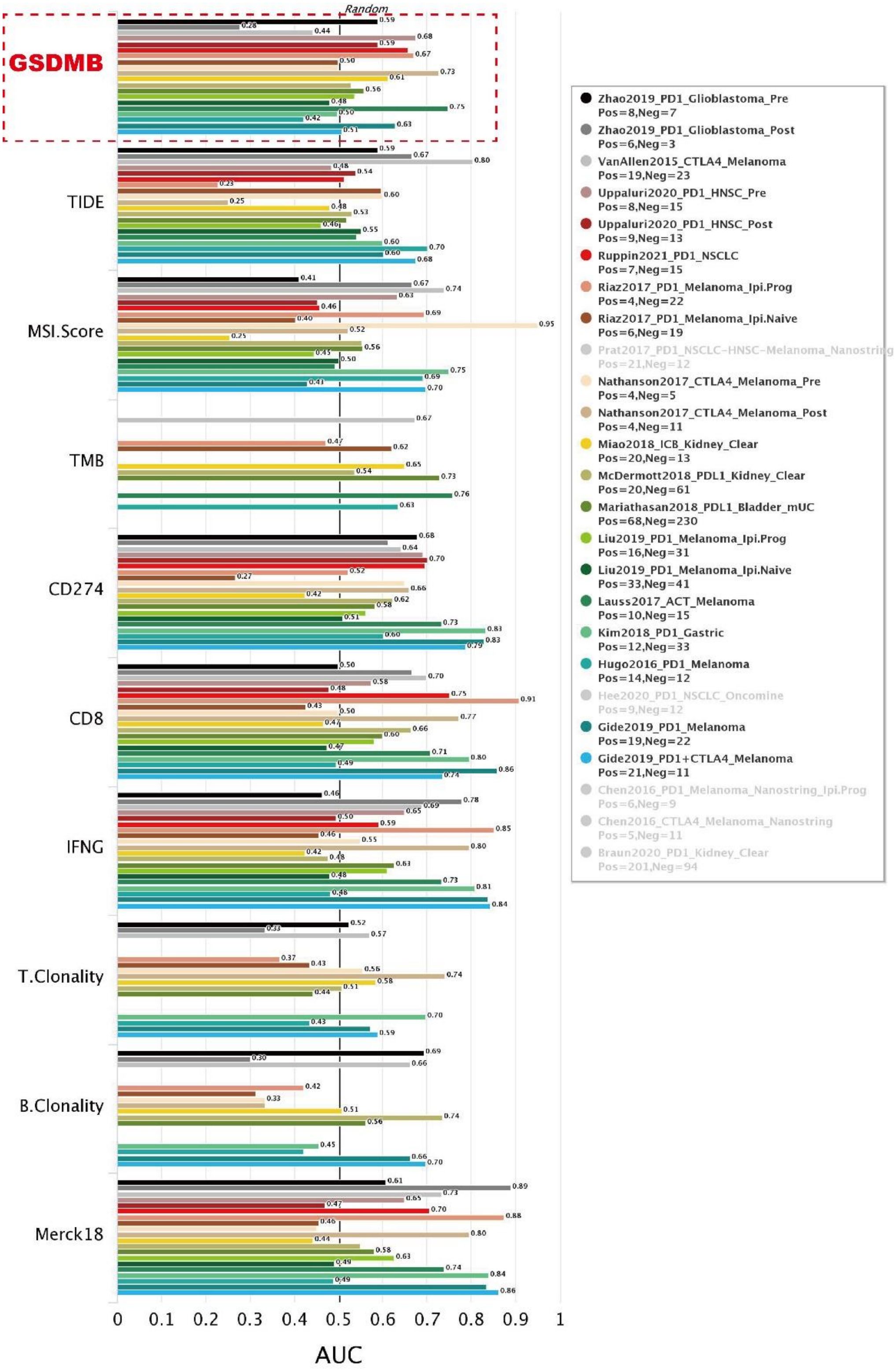

Figure S14 GSDMB expression as a predictive biomarker for immunotherapy response. The TIDE database was used to evaluate the predictive value of GSDMB expression for immunotherapy response in various cancer types. The area under the curve (AUC) was calculated for each cohort. TIDE is an algorithm that analyzes the association between gene expression and T cell function with treatment outcomes. Cohorts without GSDMB transcription data were excluded from the analysis.

Table S1 Antibodies and reagents list

Antibodies &Reagents	Cat#, Company
PE anti-human Her2 Antibody	324406, Biolegend
Alexa Fluor® 488 anti-human CD45 Antibody	304017, Biolegend
BUV395 Rat Anti-Mouse CD45 Antibody	564279, BD Biosciences
BV 421 anti-human CD3 Antibody	300434, Biolegend
APC/Cyanine7 anti-human CD4 Antibody	304014, Biolegend
PerCP/Cy5.5 anti-human CD8a Antibody（HIT8a）	300924, Biolegend
BV786 Mouse Anti-Ki-67 Antibody	563756, BD Biosciences
APC/Cyanine7 anti-human CD4 Antibody	300518, Biolegend
BV 421 anti-human CD3 Antibody	300434, Biolegend
Alexa Fluor® 488 anti-human CD107a（LAMP-1）Antibody	328610, Biolegend
Brilliant Violet 785™ anti-human CD25 Antibody	356140, Biolegend
PE T-bet Monoclonal Antibody（eBio4B10（4B10））	12-5825-82, Invitrogen
Alexa Fluor® 488 Mouse Anti-Human IFNγ	557718, BD Biosciences
Alexa Fluor®700 anti-human Granzyme A Antibody	507210, Biolegend
BD™ Cytometric Bead Array	560484, BD Biosciences
Propidium Iodide	BMS500PI, Invitrogen™
Rabbit anti-GSDMB	ab215729, Abcam
Rabbit anti-GSDMD	ab219800, Abcam
Rabbit anti-GSDME	ab215191, Abcam
Rabbit anti-PD-L1	13684, Cell Signaling Technology
Rabbit anti-β-tubulin	2146, Cell Signaling Technology
Rabbit anti-GAPDH	517<br>4, Cell Signaling Technology
Rabbit anti-Her2 (4B5)	790-4493,Ventana Roche
Mouse anti-Her2	ab265541, Abcam
Rabbit anti-Granzyme B	760-4283, Ventana Roche
Rabbit anti-Granzyme A	ab251499, Abcam
Rabbit anti-CD3	790-4341, Ventana Roche
Rabbit anti-CD8	790-4460, Ventana Roche
Rabbit anti-CD4	790-4423, Ventana Roche
Human IL-18 Precoated ELISA Kit	1121802, Dakewei
Human IL-2 Precoated ELISA Kit	1110203,Dakewei
Human IFNγ Precoated ELISA Kit	1110002, Dakewei
